# PARP14 promotes the Warburg effect in hepatocellular carcinoma by inhibiting JNK1-dependent PKM2 phosphorylation and activation

**DOI:** 10.1038/ncomms8882

**Published:** 2015-08-10

**Authors:** Valeria Iansante, Pui Man Choy, Sze Wai Fung, Ying Liu, Jian-Guo Chai, Julian Dyson, Alberto Del Rio, Clive D'Santos, Roger Williams, Shilpa Chokshi, Robert A Anders, Concetta Bubici, Salvatore Papa

**Affiliations:** 1 Cell Signaling and Cancer Laboratory, Institute of Hepatology, Foundation for Liver Research, London WC1E 6HX, UK; 2 Department of Medicine, Section of Inflammation and Signal Transduction, Imperial College, London W12 0NN, UK; 3 The Sol Goldman Pancreatic Cancer Research Center, Division of Gastrointestinal and Liver Pathology, The Johns Hopkins University School of Medicine, Baltimore, Maryland 21231, USA; 4 Department of Medical Oncology, Henan Cancer Hospital, Zhengzhou, Henan 450000, China; 5 Department of Medicine, Section of Molecular Immunology, Imperial College, London W12 0NN, UK; 6 Institute of Organic Synthesis and Photoreactivity, National Research Council, Bologna 40129, Italy; 7 Cancer Research UK Cambridge Research Institute, Li Ka Shing Centre, Cambridge CB2 0RE, UK; 8 Viral Hepatitis Laboratory, Institute of Hepatology, Foundation for Liver Research, London WC1E 6HX, UK

## Abstract

Most tumour cells use aerobic glycolysis (the Warburg effect) to support anabolic growth and evade apoptosis. Intriguingly, the molecular mechanisms that link the Warburg effect with the suppression of apoptosis are not well understood. In this study, using loss-of-function studies *in vitro* and *in vivo*, we show that the anti-apoptotic protein poly(ADP-ribose) polymerase (PARP)14 promotes aerobic glycolysis in human hepatocellular carcinoma (HCC) by maintaining low activity of the pyruvate kinase M2 isoform (PKM2), a key regulator of the Warburg effect. Notably, PARP14 is highly expressed in HCC primary tumours and associated with poor patient prognosis. Mechanistically, PARP14 inhibits the pro-apoptotic kinase JNK1, which results in the activation of PKM2 through phosphorylation of Thr365. Moreover, targeting PARP14 enhances the sensitization of HCC cells to anti-HCC agents. Our findings indicate that the PARP14-JNK1-PKM2 regulatory axis is an important determinant for the Warburg effect in tumour cells and provide a mechanistic link between apoptosis and metabolism.

## Abstract (web summary)

Tumour cells can survive by evading cell death pathways and altering their metabolism to adapt to their local environment. In this study, Iansante *et al*. show that the anti-apoptotic protein PARP14 maintains low PKM2 activity, leading to enhanced glycolysis, demonstrating a link between suppression of apoptosis and altered metabolism.

## Graphical abstract

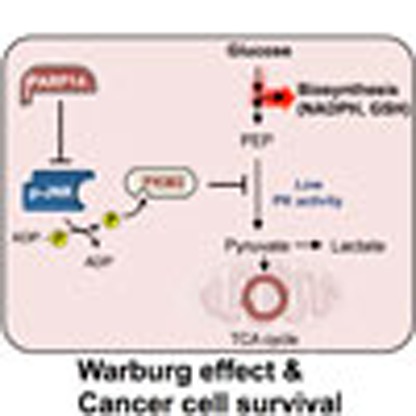

In the presence of sufficient levels of oxygen, normal quiescent cells metabolize glucose via glycolysis to pyruvate, which is further metabolized through mitochondrial oxidative phosphorylation for ATP production, whereas under hypoxic/anaerobic conditions, cells ferment glucose to lactate^1^,^2^. In contrast, rapidly dividing cells convert much of the glucose into lactate irrespective of oxygen availability. This cell metabolism, known as aerobic glycolysis or the Warburg effect^1^,^2^, allows dividing cells to use intermediary glucose metabolites to generate reducing equivalents (such as NADPH) and macromolecules (such as nucleotides, proteins and lipids) required for the doubling of biomass, and to suppress apoptosis^3^,^4^. As such, increased aerobic glycolysis is a widely observed feature in human cancers and often correlates with tumour aggressiveness and poor patient prognosis in many tumour types, including human hepatocellular carcinoma (HCC), the main type of liver cancer^5^,^6^,^7^,^8^. Accumulating evidences indicate that the Warburg effect occurs downstream of pro-survival signalling pathways that are altered by loss of tumour suppressors or activation of oncogenes, such as p53 and Myc^4^,^9^,^10^,^11^,^12^. Importantly, interference with altered metabolism in tumour cells results in reduced tumourigenicity and increased apoptotic sensitivity to chemotherapeutics^3^,^4^,^13^,^14^, suggesting that the aerobic glycolytic metabolism is central to tumour growth and survival^2^,^3^,^4^. Thus, a better understanding of the mechanistic links between cell metabolism and survival control could be of paramount significance for the development of new therapeutics particularly in HCC, given the close association between metabolic alteration and HCC pathogenesis^5^,^6^,^7^,^14^. Poly(ADP-ribose) polymerase (PARP)14, a member of the PARP family of proteins^15^, is a well-established pro-survival protein that has been involved in protecting lymphocytes against apoptosis^16^,^17^. PARP14 has been also shown to accelerate lymphomagenesis driven by persistent overexpression of the oncogene c-Myc, and to be vital for interleukin-4-induced glycolytic flux in mice^16^,^17^, indicative of a potential link between PARP14 and metabolic regulation. In human fibrosarcoma cells, PARP14 was shown to regulate the abundance of phosphoglucose isomerase, a glycolytic enzyme involved in tumour aggressiveness^18^. In addition, we have recently shown that PARP14 is essential for survival of human multiple myeloma cells and that PARP14 levels positively correlate with disease progression and poor prognosis^19^. We have also shown that the pro-survival function of PARP14 depends on the suppression of the apoptotic signalling mediated by JNK1, a highly conserved serine/threonine protein kinase^19^,^20^. However, whether the survival function of PARP14 contributes to the Warburg effect in human cancer cells have not been previously investigated.

A critical mediator of the Warburg effect is pyruvate kinase M2 isoform (PKM2), a tumour-specific isoform of the glycolytic enzyme pyruvate kinase (PK), which catalyses the synthesis of pyruvate and ATP, using phosphoenolpyruvate (PEP) and ADP as substrates^21^,^22^,^23^,^24^. PKM2, but not its spliced variant PKM1, has a low PK activity that favours the Warburg effect and provides advantages for cancer cell growth and survival^20^,^24^,^25^. In contrast, high PK activity or pharmacological activation of PKM2 was shown to inhibit aerobic glycolytic phenotypes and tumour growth^26^,^27^,^28^, underscoring the importance of identifying endogenous regulators of PKM2 activity in cancer.

Here we show that PARP14 is highly expressed in diverse solid tumours, including HCC, and positively regulates aerobic glycolysis through effects on PKM2 enzymatic activity, thus promoting survival in cancer cells. We demonstrate that PARP14 maintains low PKM2 activity by inactivation of JNK1, which, in turn, mediates the phosphorylation of PKM2 at Thr365; phosphorylation of PKM2 at this residue increases PKM2 activity. Thus, this study reveals a molecular link between a regulator of cell survival and a key enzyme in cancer metabolism with potential therapeutic implications.

## Results

### PARP14 is upregulated in HCC and cirrhotic livers

**Figure 1:**
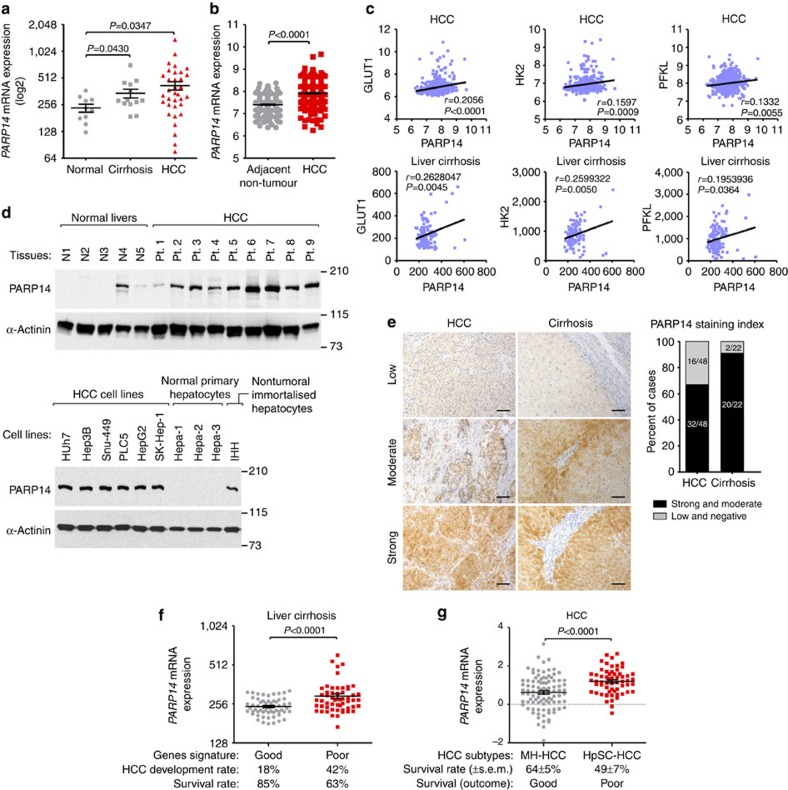
PARP14 is upregulated in cirrhotic and HCC livers and associated with glycolytic gene expression and poor patient prognosis. (**a**) Gene expression levels of human *PARP14* mRNA (NM_017554) in normal quiescent (*n*=10), cirrhotic (*n*=13) and HCC (*n*=35) livers (clinical data set GSE6764; ref. 34). The horizontal lines indicate mean±s.e.m. *P* values were calculated by Student's *t*-test. (**b**) Levels of *PARP14* mRNA in HCC (*n*=240) and adjacent nontumour (*n*=193) tissue (GSE36376; ref. 35). The horizontal lines indicate mean±s.e.m. *P* values were calculated by nonparametric Mann–Whitney tests. (**c**) Scatterplots showing the positive correlation between *PARP14* and *GLUT1/HK2/PFKL* mRNA expression in HCC (top; GSE36376) and cirrhotic patients (bottom; GSE15654; ref. 38). Pearson's coefficient tests were performed to assess statistical significance. (**d**) Western blots (WBs) analyses detecting PARP14 in lysates of normal quiescent livers, HCC biopsies, HCC-derived cell lines, primary normal hepatocytes isolated from three healthy livers and nontumoural immortalized human hepatocytes (IHH) (refs 36, 37). α-Actinin was used as loading control. (**e**) PARP14 immunostaining of tissue microarray comprising 48 HCC and 22 cirrhotic livers. Shown are representative images of the immunostainings at 20 × magnification. Scale bar, 50 μm. Graph indicates the percentage of cases displaying strong or low staining intensity of PARP14. (**f**) Levels of *PARP14* transcripts in the clinical data set GSE15654 consisting of 115 patients with newly diagnosed cirrhosis who were prospectively followed up in an HCC surveillance program and classified as having good (*n*=55) and poor (*n*=60) prognosis based on the rates of patient survival and incidence of developing HCC. (**g**) Scatterplots showing *PARP14* mRNA expression in two molecular subtypes of HCC, mature hepatocyte-like HCC (MH-HCC; *n*=96; EpCAM− and AFP−) and HpSC-HCC (*n*=60; EpCAM+ and AFP+) (ref. 39). (**f**,**g**) Table indicates a summary of the clinical parameters associated with each group. The horizontal lines indicate mean±s.e.m. *P* values were calculated by nonparametric Mann–Whitney tests.

As an initial attempt to explore whether PARP14 regulates the Warburg effect, we evaluated the expression of PARP14 transcripts in highly glycolytic liver samples^5^,^6^,^7^,^29^,^30^,^31^,^32^,^33^ from patients with HCC and patients with cirrhosis, a liver condition that predisposes patients to HCC^7^,^29^,^34^, by interrogating public gene expression databases. We found that in HCC and cirrhotic samples, the expression of PARP14 was significantly higher than that in normal quiescent liver samples (GSE6764)^34^ (Fig. 1a). In a second, independent data set (GSE36376)^35^, we also observed significantly higher expression of PARP14 in HCC samples compared with their adjacent nontumour tissues (Fig. 1b). Importantly, the expression levels of PARP14 in HCC and cirrhotic livers positively correlated with expression of key glycolytic genes (Fig. 1c), suggesting a possible role for PARP14 in the glycolytic phenotype of highly proliferating cirrhotic and malignant hepatocytes^5^,^6^,^7^,^29^,^30^,^31^,^32^,^33^. To confirm these observations experimentally, we carried out western blots (WBs) in a panel of HCC-derived cell lines (Huh7, Hep3B, Snu-449, PLC5, HepG2 and SK-Hep-1) and tumour lysates obtained from nine patients with HCC. While only one of five normal livers displayed PARP14 expression, the expression of PARP14 was higher in eight of nine HCC samples and in all HCC cell lines examined (Fig. 1d). Interestingly, nontumoural immortalized human hepatocyte (IHH) cell line^36^,^37^ and three independent primary normal hepatocytes displayed low to undetectable levels of PARP14 protein (Fig. 1d). These findings were further supported by immunohistochemical analyses in primary HCCs (*n*=48) and cirrhotic (*n*=22) livers. Of HCC cases, 32 out of 48 (67%) were strong or moderately positive for PARP14 and 16 (33%) were weak or negative (Fig. 1e). Moderate or strong immune positivity for PARP14 was also present in 20 out of 22 cirrhotic cases, suggesting that PARP14 is involved in the tumourigenesis of HCC. In support of this hypothesis, high PARP14 mRNA expression levels were found to be associated with a progression of cirrhosis to HCC and reduced survival rate in a cohort of 115 newly diagnosed cirrhotic patients (GSE15654)^38^ (Fig. 1f). Remarkably, PARP14 is overexpressed in early stages of HCC and, importantly, its high expression is maintained during cancer progression, indicating that PARP14 upregulation might be required for both the formation and maintenance of HCC (Supplementary Fig. 1a). To gain additional clinical insights, we evaluated the expression of PARP14 in molecular subtypes of HCC with distinct prognoses^39^ using a publicly available data set of 156 HCC cases. PARP14 expression was significantly higher in the poor prognostic hepatic stem cell-like HCC (HpSC-HCC) subtype than in the mature hepatocyte-like HCC subtype of HCC (Fig. 1g)^39^. Consistent with the hypothesis that PARP14 is involved in hepatocarcinogenesis, HpSC-HCC subtype has tumour-initiating features^39^. Finally, the analysis of additional data sets also showed that PARP14 expression was higher in glioblastoma, breast, gastric and lung cancers compared with that in their matched normal tissue (Supplementary Fig. 1b). This is in full agreement with the notion that the Warburg effect is a common characteristic of human carcinomas^2^,^8^. Collectively, these data suggest that PARP14 may play an oncogenic role in multiple cancer types.

### PARP14 is required for tumour growth

**Figure 2:**
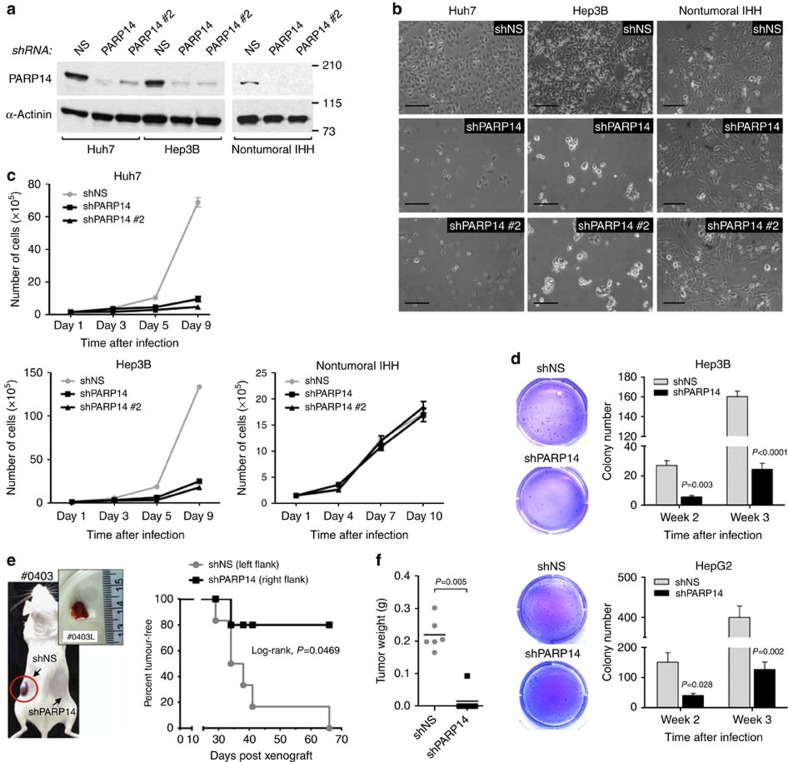
PARP14 knockdown impairs HCC cell growth. (**a**) WBs showing the PARP14 knockdown efficiency of Huh7, Hep3B and non-malignant IHH cells stably expressing PARP14, PARP14#2 or control nonspecific (NS) shRNAs. (**b**) Shown are representative light microscopy images of cultured cells (days 5 and 7 after shRNA expression). Scale bar, 2 μm. (**c**) Growth curves of Huh7 (*n*=6), Hep3B (*n*=6) and non-malignant IHH (*n*=6) cells stably expressing PARP14, PARP14#2 or control nonspecific (NS) shRNAs. Data are shown as mean±s.e.m. and are representative of three independent cultures. (**d**) Time course of anchorage-independent colony formation by Hep3B and HepG2 cells stably expressing shPARP14 or shNS. Representative images show overall view of colony growth after 3 weeks. Data shown are mean±s.e.m. of three independent cultures. *P* values were calculated by Student's *t*-test. (**e**) Kaplan–Meier curve showing tumour formation over time of Huh7 cells expressing shPARP14 and shNS injected subcutaneously in the right and left flanks of NOD/SCID immunodeficient mice (*n*=6), respectively. *P* value was calculated by Log-rank test. Image of a visible tumour developed in the flank (framed in a circle) of a representative mouse. The inset shows the size of the tumour explanted from the left flank. (**f**) Tumour weights were analysed by scatterplot. The horizontal lines indicate the mean. *P* value indicates Student's *t*-test.

To assess the functional role of PARP14 in cancer cells, we knocked down expression of PARP14 in HCC cells using two PARP14-specific short hairpin (sh)RNA lentiviruses (shPARP14 or shPARP14#2). As controls, we used lentiviruses expressing nonspecific shRNA (shNS)^19^. Efficient PARP14 knockdown was confirmed by WB (Fig. 2a). We found that PARP14 shRNA-expressing cells had a significantly slower growth rate and a reduced ability to grow in an anchorage-independent manner compared with control cells (Fig. 2b–d), indicating that PARP14 depletion confers a growth disadvantage. By contrast, depletion of PARP14 did not affect the growth rate of nontumoural IHH cells (Fig. 2b,c). Next, we subcutaneously injected Huh7 cells bearing PARP14 or NS shRNAs into NOD/SCID immunodeficient mice to assess the ability of these cells to form tumours *in vivo*. Large tumours developed in every site injected with shNS-transduced cells after a median time of 36 days (Fig. 2e,f; Supplementary Fig. 2a). Only one tumour developed from shPARP14-transduced cells was smaller in size and had partially regained PARP14 expression (Fig. 2f; Supplementary Fig. 2a,b). Thus, PARP14 is required for HCC cell growth both *in vitro* and *in vivo*. Of clinical relevance, dose-response analyses revealed that knockdown of PARP14 rendered HCC cells more sensitive to sorafenib or doxorubicin (Supplementary Fig. 2c), two anti-HCC agents 40, suggesting an additive effect. Such an effect was also observed when we silenced PARP14 in other cancer cell types and treated them with doxorubicin (Supplementary Fig. 2d). Notably, PARP14 depletion had no significant effect on nontumoural IHH cells treated with either sorafenib or doxorubicin (Supplementary Fig. 2c).

### Knockdown of PARP14 impairs the Warburg effect

**Figure 3:**
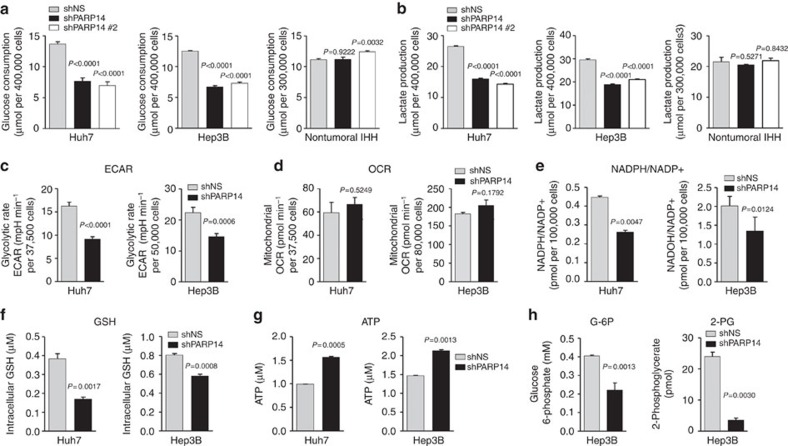
PARP14 is critical for HCC glycolytic activity. (**a**,**b**) Glucose consumption (**a**) and lactate production (**b**) in HCC and non-malignant IHH cells stably expressing PARP14, PARP14#2 or control NS shRNAs (day 4 after shRNA expression). (**c**,**d**) ECAR (a proxy for the rate of glycolysis) (**c**) and OCR (a proxy for mitochondrial respiration) (**d**) in Huh7 and Hep3B cells stably expressing PARP14 or NS shRNAs. (**e**–**g**) Reduced levels of NADPH/NADP+ (**e**) and glutathione (GSH) (**f**), and increased levels of ATP (**g**) in shPARP14-expressing Huh7 and Hep3B cells compared with control shNS-expressing cells. (**a**–**g**) Data shown are mean±s.e.m. of *n*≥3 technical replicates and are representative of three independent experiments. *P* values were calculated by Student's *t*-test. (**h**) Reduced levels of glucose-6-phosphate (G6P) and 2-phosphoglycerate (2-PG) in shPARP14-expressing Hep3B cells compared with control shNS-expressing cells. Data shown are mean±s.e.m. of two independent cultures. *P* values were calculated by Student's *t*-test.

To understand the mechanisms underlying the tumourigenic function of PARP14, we examined whether PARP14 affected aerobic glycolysis. Compared with control silencing, knockdown of PARP14 in multiple HCC cell lines resulted in decreased glucose consumption and lactate production (Fig. 3a,b; Supplementary Fig. 3a,b), accompanied by a reduced rate of extracellular acidification (ECAR) (Fig. 3c; Supplementary Fig. 3c), indicators of decreased aerobic glycolysis^2^,^4^,^8^. Notably, PARP14 silencing did not result in a significant change of oxygen consumption rate (OCR) or activity of the mitochondrial complex I (Fig. 3d; Supplementary Fig. 3d,e), indicating that depletion of PARP14 attenuates glycolysis without affecting mitochondrial oxidative phosphorylation^2^.

Furthermore, we observed a significant decrease of NADPH, a crucial metabolite for reductive biosynthesis of macromolecules^2^,^27^,^41^, in PARP14 knockdown cells that correlated with a significant decrease of gluthatione (GSH) and increase in ATP levels (Fig. 3e–g). Accordingly, compared with control cells, PARP14-depleted cells accumulated less glucose-6-phosphate and 2-PG, two key glycolytic intermediates involved in nucleotide and serine synthesis (Fig. 3h)^2^,^4^,^42^. This suggests that PARP14 may favour biomass generation over energy production, a metabolic shift consistent with the Warburg effect^1^,^2^,^4^. Importantly, knocking down PARP14 also impaired aerobic glycolytic metabolism in other highly glycolytic human cell lines derived from multiple myeloma (RPMI-8226), brain (U87) and breast (MCF7) cancers (Supplementary Fig. 3f,g)^43^,^44^,^45^. By contrast, PARP14 knockdown in nontumoural IHH cells had no effect on glucose consumption or lactate production (Fig. 3a,b). Moreover, levels of PARP14 protein decreased in a time- and dose-dependent manner when the oncoprotein Myc, one of the central regulators of aerobic glycolysis in most types of tumours^10^,^11^,^12^, was inhibited in hepatoma cells (Supplementary Fig. 3h). These results suggest that PARP14 may be a universal regulator of aerobic glycolysis in human cancers.

### PARP14 inhibits the metabolic activity of PKM2

**Figure 4:**
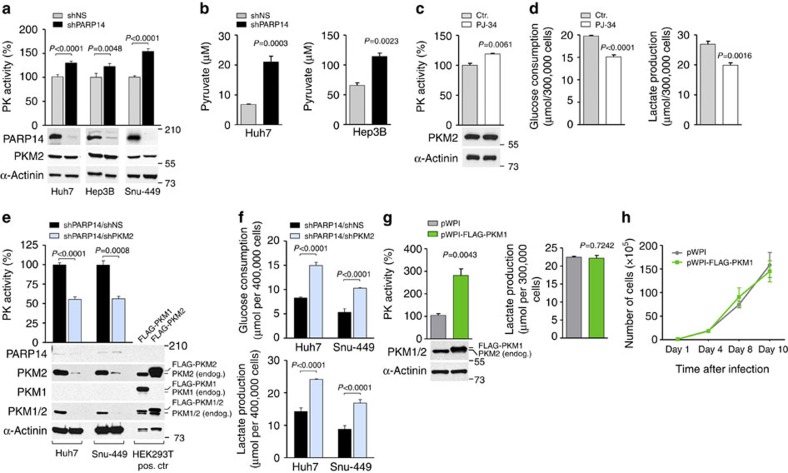
PARP14 inhibits PKM2 activity to promote the Warburg effect. (**a**) Pyruvate kinase (PK) enzymatic activity in lysates of Huh7, Hep3B and Snu-449 cells stably expressing shNS or shPARP14. WBs showing the levels of PARP14 and PKM2 proteins in matching cell lysates. (**b**) Quantified intracellular pyruvate concentrations in control and shPARP14 HCC cells (Huh7 and Hep3B cells). (**c**) PK enzymatic activity in lysates of Hep3B cells left untreated (ctr.) or treated with 10 μM PJ-34 for 48 h. WBs showing the levels of endogenous PKM2 in matching cell lysates. (**d**) Glucose consumption and lactate production in Hep3B cells left untreated (ctr.) or treated with 10 μM PJ-34 for 48 h. (**e**) PK enzymatic activity in PARP14-depleted HCC cells co-expressing either PKM2 (shPARP14/shPKM2) or control NS (shPARP14/shNS) shRNAs. WBs analyses with antibodies against endogenous (endog.) proteins in co-silenced HCC cells used for the corresponding assay. Lysates of HEK293T cells overexpressing FLAG-PKM1 or FLAG-PKM2 were used as positive controls (pos. ctr). (**f**) Glucose consumption and lactate production in Huh7 and Snu-449 co-expressing shPARP14/shNS or shPARP14/shPKM2. (**g**) PK enzymatic activity and lactate production in Hep3B cells stably expressing FLAG-PKM1 (pWPI-FLAG-PKM1) or control empty vector (pWPI). WBs showing the levels of endogenous PKM2 and exogenous PKM1 in cell lysates. (**h**) Growth curves of Hep3B cells stably expressing FLAG-PKM1 (pWPI-FLAG-PKM1) or control empty vector (pWPI). (**a**–**h**) Data shown are mean±s.e.m. of *n*≥3 technical replicates and are representative of at least three independent experiments. *P* values were calculated by Student's *t*-test.

As a constitutively low PKM2 activity is essential for aerobic glycolysis^25^,^27^,^28^,^46^, we investigated a possible relationship between PKM2 and PARP14. First, we examined the effect of PARP14 on PK activity and found that, compared with control shNS-infected cells, knockdown of PARP14 in HCC cells significantly increased PK activity with a corresponding marked increase in pyruvate levels (Fig. 4a,b). Similarly, activation of PK was also induced by the pan-PARP inhibitor PJ-34, which has been previously used to examine PARP14 function (Fig. 4c)^17^,^19^,^47^. This effect was accompanied with reduced aerobic glycolysis (Fig. 4d). To determine whether the increase in PK activity is attributable to PKM2 and responsible for impaired glycolysis caused by PARP14 knockdown, we co-expressed PKM2 shRNA (shPKM2) in PARP14-depleted cells (Fig. 4e) and determined the possibility of rescuing the metabolic effects observed in these cells. PK assays confirmed that total PK activity level was substantially reduced in the double PARP14/PKM2 knockdown cells compared with control cells, supporting the hypothesis that intracellular PK activity is mostly likely due to the predominant expression of PKM2 in HCC cells. Of note, no compensatory increase in the constitutively active splice variant PKM1 (refs 1, 21, 27) was observed in the shPARP14/shPKM2 cells, although PKM2 expression was almost completely abolished (Fig. 4e). Depletion of PKM2 was sufficient to completely reverse decreased aerobic glycolysis caused by PARP14 depletion (Fig. 4f). The effect of co-silencing PKM2 with PARP14 on glucose consumption and lactate production was comparable to that of control shNS-infected cells (Figs 3a,b and 4f; compare shPARP14/shPKM2 with shNS). These effects were specific, as control silencing did not affect PARP14-depleted cells. Remarkably, ectopic expression of PKM1 in PKM2-expressing cells was sufficient to increase their total PK activity, but had no effect on lactate production and cell proliferation in standard tissue culture conditions (Fig. 4g,h). Together, these results indicate that the impaired aerobic glycolysis observed in PARP14-depleted cells is a consequence of PKM2 activation.

PKM2 can translocate to the nucleus, where it collaborates with transcription factors, most notably, hypoxia-inducible factor (HIF)1α and Myc, in inducing glycolytic gene expression that promotes the aerobic glycolysis in cancer cells^10^,^11^,^24^,^48^. Thus, we asked whether PARP14 could also affect the nuclear function of PKM2. A slight but significant decrease in the nuclear levels of PKM2 was observed in PARP14-depleted cells compared with the levels of nuclear PKM2 in control shNS-infected cells (Supplementary Fig. 4a). In line with this finding, PARP14 knockdown cells also displayed impaired transcriptional activity of HIF1α and Myc as well as the consequent protein expression of glycolytic enzymes (Supplementary Fig. 4b–d), a metabolic signature consistent with an impaired aerobic glycolysis^2^,^10^,^11^,^24^,^48^.

**Figure 5:**
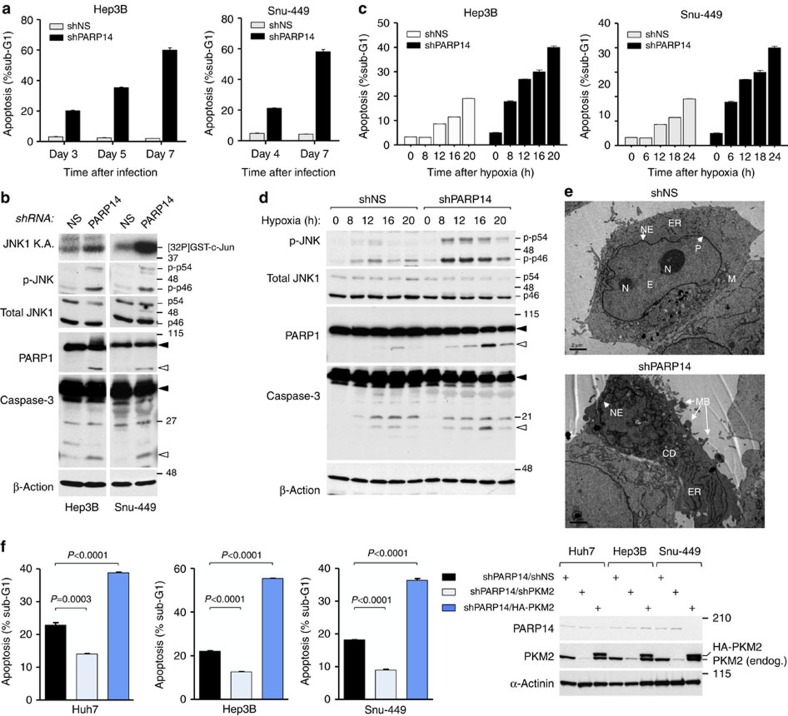
PARP14 promotes HCC cell survival by inhibiting PKM2. (**a**–**d**) Analyses of shNS- and shPARP14-expressing Hep3B and Snu-449 cells cultured at the indicated time under normal (**a**,**b**) or hypoxic conditions (**c**,**d**) showing the percentage of apoptosis in the population sub-G1 (DNA content) (**a**,**c**) and the levels of apoptotic markers (**b**,**d**) by WBs. Closed and open arrowheads indicate the pro-cleaved and cleaved (active) products of the indicated proteins, respectively. p54 and p46 denote the JNK splicing isoforms. *In vitro* JNK1 kinase activity (JNK1 KA) was performed using glutathione S-transferase c-Jun as substrate in the presence of [32P]-γ-ATP. (**e**) Morphological assessment of apoptosis by electron microscopy in Hep3B cells expressing control shNS versus shPARP14. shNS-expressing Hep3B cells exhibit an homogenous nuclear envelop (NE), distinct nucleoli (N), euchromatin (E) associated with the nuclear pores (P), normal distribution of endoplasmic reticulum (ER) and mitochondria (M). shPARP14-expressing Hep3B cells displays early signs of apoptosis, such as clear structural alteration of the shape and surface of the cells, cytoplasmic disorganization (CD) with the presence of poorly defined ER and organelles, non-homogenous NE and peripheral membrane blebs (MB). Scale bar, 2 μm. (**f**) Percentage of apoptosis in PARP14-depleted HCC cells co-expressing either shPKM2 or HA-PKM2. WBs analyses showing endogenous (endog.) and ectopic expression of indicated proteins. (**a**,**c**,**f**) Data shown are mean±s.e.m. of three independent cultures. *P* values were calculated by Student's *t*-test.

As inactivation of PKM2 has been associated with the growth and survival of multiple cancer cells^21^,^26^,^27^,^28^,^46^, we next examined the impact of PARP14 on the survival of HCC cells. A progressive increase of apoptosis in PARP14 knockdown cells was observed over time, as shown by a marked increase in the sub-G1 population and caspase-3 and PARP1 cleavage (Fig. 5a,b; Supplementary Fig. 5a–c). These effects of PARP14 knockdown were also observed in HCC cells exposed to hypoxia (1% oxygen), which strongly triggers aerobic glycolysis^8^,^24^ (Fig. 5c,d). Electron microscopy images also confirmed that PARP14-depleted Hep3B cells underwent characteristic morphological changes (that is, shrinking and blebbing) indicative of apoptosis (Fig. 5e). As expected, in IHH cells, PARP14 depletion did not induce apoptosis (Supplementary Fig. 5b), confirming that PARP14 is not essential for basal survival of nontumoural IHH cells. Notably, PARP14 knockdown cells displayed increased phosphorylation of the pro-apoptotic kinase JNK1 (predominantly present as p46 isoform) compared with control shNS cells (Fig. 5b,d; Supplementary Fig. 5c), indicating that depletion of PARP14 increased the basal activity of JNK1, thereby resulting in apoptosis. Activation of JNK1 was further confirmed by *in vitro* kinase assays (JNK1 KAs; Fig. 5b; Supplementary Fig. 5c). Similarly, a dose- and time-dependent JNK1 activation and apoptosis were also induced by PJ-34 (Supplementary Fig. 5d,e). Of clinical relevance, a significant inverse correlation between PARP14 and p-JNK levels was observed in patient-derived lysates of HCC (Supplementary Fig. 5f; see also Fig. 1d), strongly suggesting that PARP14-mediated JNK1 suppression occurs in HCC. To assess whether apoptosis triggered by PARP14 knockdown was mediated by PKM2 activation, we either silenced or overexpressed PKM2 in PARP14-depleted HCC cells. Whereas knockdown of PKM2 reversed apoptosis induced by PARP14 depletion, PKM2 ectopic expression increased apoptosis by 50% (Fig. 5f). Hence, PARP14 promotes the Warburg effect needed for HCC cell survival by lowering PKM2 activity.

### PARP14 inhibits PKM2 activity through inactivation of JNK1

**Figure 6:**
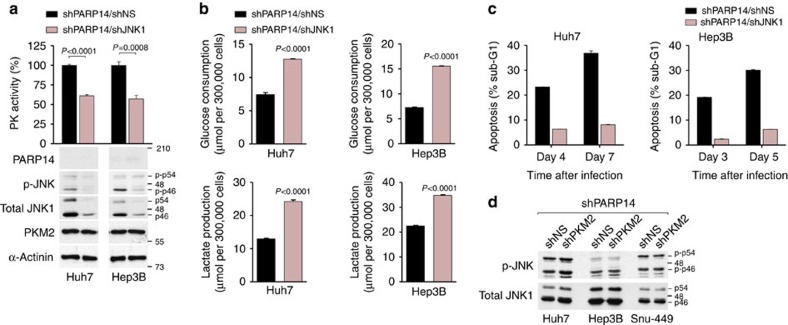
PARP14 inhibits PKM2 activity via suppression of JNK1. (**a**,**b**) PK activity (**a**), glucose consumption and lactate production (**b**) were analysed in PARP14-depleted Huh7 and Hep3B cells co-expressing either JNK1 (shPARP14/shJNK1) or control nonspecific (shPARP14/shNS) shRNAs. WBs analyses (**a**, bottom panels) showing knockdown efficiency of PARP14 and JNK1. Data shown are mean±s.e.m. of *n*=4 replicates and are representative of at least three independent experiments. *P* values were calculated by Student's *t*-test. (**c**) Percentage of apoptosis in Huh7 and Hep3B co-expressing shPARP14/shNS or shPARP14/shJNK1. Data shown are mean±s.e.m. of three independent cultures. (**d**) WBs showing levels of phospho-active JNK (p-JNK) in HCC cells co-expressing shPARP14/shPKM2 or shPARP14/shNS. Total JNK1 serves as loading control.

We then investigated the mechanisms of PKM2 inhibition by PARP14. The observations that JNK1 is activated by PARP14 inhibition (see Fig. 5b,d; Supplementary Fig. 5c,d) and negatively regulates hepatic glycolysis^49^ led us to examine whether activation of JNK1 mediated the effects of PARP14 on PKM2 activity. For this purpose, we knocked down JNK1 expression in HCC cells in combination with PARP14 using JNK1 shRNA (shJNK1) and assayed for the PKM2 enzyme activity. Knocking down JNK1 prevented the increase in PKM2 activity in PARP14-depleted cells (Fig. 6a), showing that JNK1 is responsible for PKM2 activation in these cells. In parallel, we observed that co-depletion of JNK1 with PARP14 completely rescued the reduced glucose consumption and lactate production as well as apoptotic phenotype associated with PARP14 knockdown (Fig. 6b,c). Remarkably, no significant differences in phosphorylation/activity levels of JNK1 were observed when PKM2 was silenced in combination with PARP14 (Fig. 6d), which is consistent with the hypothesis that JNK1 functions upstream of PKM2. These results show that, by suppressing JNK1, PARP14 inhibits PKM2 activity.

**Figure 7:**
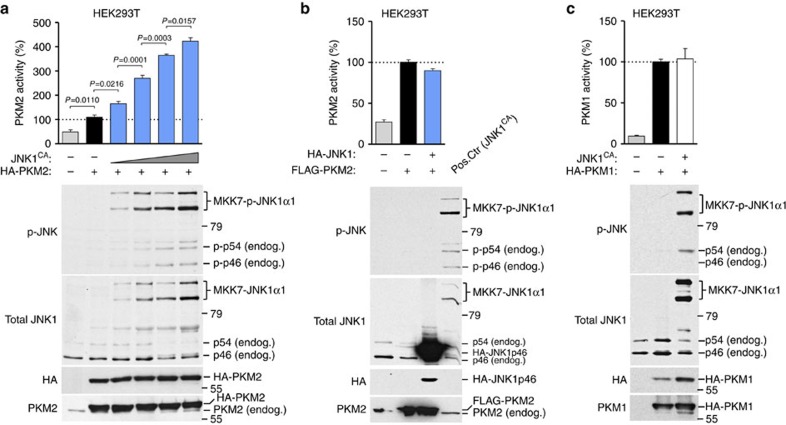
Active JNK1 specifically activates PKM2 but not PKM1. (**a**,**b**) PKM2 activity was assessed in lysates of HEK293T cells transfected with HA-PKM2 (10 μg) in combination with increasing amounts of constitutive active JNK1 (JNK1^CA^; 2.5, 5, 10 and 20 μg) or control empty vector (−) (**a**) or with FLAG-PKM2 (15 μg) and non-active HA-JNK1 (15 μg) (**b**). Lysates of HEK293T cells overexpressing JNK1^CA^ were used as positive controls (pos. ctr) for the detection of phospho-active JNK (p-JNK). WB analyses showing levels of phosphorylated endogenous (endog.) JNK1 and JNK1^CA^ (MKK7-JNK1α1), and HA and PKM2. Levels of total JNK1 serve as loading control. (**c**) PKM1 enzymatic activity was assessed in lysates of HEK293T cells transfected with equal amounts of HA-PKM1 (15 μg) and JNK1 constitutive active (JNK1^CA^; 15 μg) or control empty vector (−; 15 μg). WBs analyses showing levels of phosphorylated endogenous (endog.) JNK1 and JNK1^CA^ (MKK7-JNK1α1), and HA and PKM1. (**a**–**c**) Data shown are mean±s.e.m. of three biological replicates for all assays. *P* values were calculated by one-way analysis of variance (*P*<0.0001) followed by Bonferroni's multiple comparison tests.

To further examine the effects of JNK1 on PKM2 activity, we co-expressed increasing amounts of a constitutively active form of JNK1 (JNK1^CA^)^50^ in HEK293T cells with HA-tagged PKM2 and measured PKM2 activity in the corresponding cell lysates. Expression of JNK1^CA^ significantly increased PKM2 activity in a dose-dependent manner (Fig. 7a). Such an effect was not observed when we co-expressed a catalytically non-active JNK1 protein (Fig. 7b), suggesting that the kinase activity of JNK1 may be required for PKM2 activation. Moreover, when JNK1^CA^ was co-expressed with PKM1 isoform, PKM1 activity was unaffected (Fig. 7c), indicating that active JNK1 specifically stimulates PKM2.

### JNK1 binds to and activates PKM2 through phosphorylation

**Figure 8:**
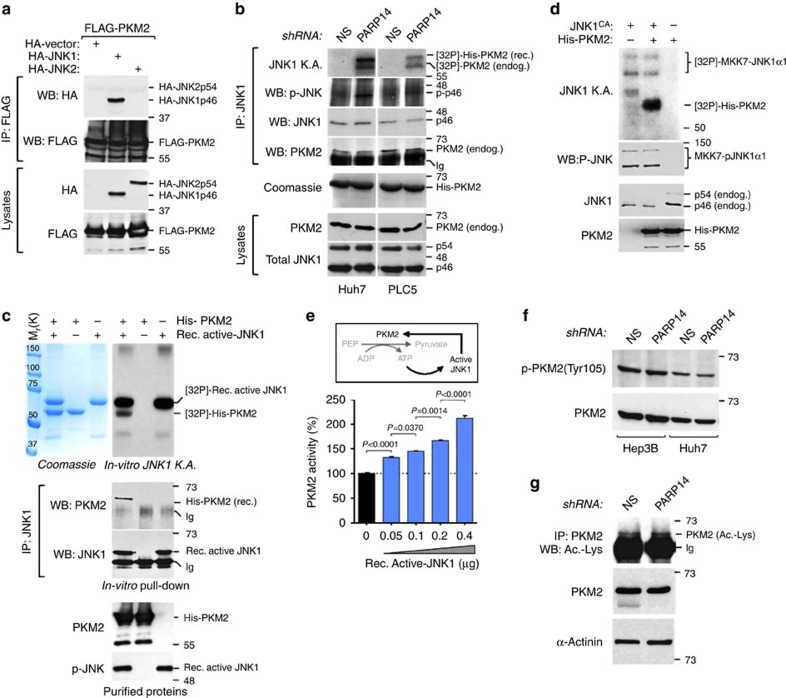
JNK1 interacts with and activates PKM2 by phosphorylation. (**a**) Protein lysates of HEK293T cells transfected with FLAG-PKM2 in combination with HA-JNK1, HA-JNK2 or empty vector were subject to immunoprecipitation (IP) with anti-FLAG antibody followed by WBs analyses as indicated. (**b**) Activated JNK1 was immunoprecipitated (IP:JNK1 and WB:p-JNK) from lysates of Huh7 and PLC5 cells expressing nonspecific (shNS) or PARP14 (shPARP14) shRNAs and assayed for kinase activity (KA) using recombinant (rec.) His-PKM2 as substrate in the presence of [32P]-γ-ATP. [32P]-PKM2 (endog.) denotes the phosphorylation of endogenous PKM2, which was co-immunoprecipitated with JNK1 from the same lysates (IP:JNK1 and WB:PKM2). (**c**) *In vitro* JNK1 KA was performed by incubating recombinant activated JNK1 (rec. active JNK1) with His-PKM2 as substrate. [32P]-Rec. active JNK1 denotes autophosphorylation. *In vitro* pull-down assays were performed by IP and WBs after incubating purified rec. active JNK1 and His-PKM2 (IP:JNK1 and WB:PKM2). Coomassie staining shows the purity and size of the recombinant proteins. (**d**) Activated JNK1 was immunoprecipitated (IP:JNK1 and WB:p-JNK) from lysates of HEK293T cells expressing JNK1 constitutive active (JNK1^CA^) and assayed for kinase activity (KA) using recombinant His-PKM2 as substrate in the presence of [32P]-γ-ATP. [32P]-MKK7-JNK1α1 denotes the autophosphorylation of transfected JNK1^CA^. (**e**) PKM2 activity was evaluated mixing recombinant His-PKM2 protein with different amounts of purified Rec. active JNK1 in the presence of PEP and ADP. The resultant formation of ATP serves as an internal catalyst for the *in vitro* JNK1-mediated phosphorylation/activation of PKM2 (top scheme). Data shown are mean±s.e.m. of three biological replicates. *P* values were calculated by one-way analysis of variance (*P*<0.0001) followed by Bonferroni's multiple comparison tests. (**f**) WBs showing levels of phospho-PKM2(Tyr105) (p-PKM2(Tyr105)) in HCC cells expressing shPARP14 or control shNS. Total PKM2 serves as loading control. (**g**) Immunoprecipitation (IP) of PKM2 followed by WBs analyses detecting acetylated lysine (ac.-lys) in Hep3B cells expressing shPARP14 or control shNS. Total PKM2 and α-actinin were used as loading control. Ig, immunoglobulin.

To determine the molecular mechanism of how JNK1 activates PKM2, we investigated whether JNK1 interacts with PKM2. FLAG-tagged PKM2 or FLAG-PKM1 was co-expressed in HEK293T cells with HA-JNK2, HA-JNK1 or HA-empty vector, and protein associations were assessed by combined immunoprecipitations (IPs) and WB analyses. HA-JNK1 specifically bound to FLAG-PKM2, but not to FLAG-PKM1 (Fig. 8a; Supplementary Fig. 6a). The lack of interaction of the closely related HA-JNK2 (ref. 19) with PKM2 further confirmed the specificity of binding (Fig. 8a). Similarly, endogenous JNK1 interacted with endogenous PKM2 in HCC cells (Fig. 8b; IP:JNK1 and WB:PKM2). The binding of JNK1 to PKM2 is direct, as shown by pull-down analyses with purified recombinant proteins (Fig. 8c; IP:JNK1 and WB:PKM2). To determine whether JNK1 could phosphorylate PKM2, we performed immune complex kinase assays and revealed that shPARP14-activated JNK1 markedly phosphorylated both purified His-PKM2 and endogenous PKM2 in HCC cells (Fig. 8b; JNK1 KA). Similar results were observed in HEK293T cells ectopically expressing JNK1^CA^ (Fig. 8d; JNK1 KA). Consistent with their direct interaction, active recombinant JNK1 phosphorylated purified His-PKM2, but not His-PKM1 (Fig. 8c; Supplementary Fig. 6b; *in vitro* JNK1 KA). Altogether, these data indicate that PKM2 is a direct substrate of JNK1. Importantly, phosphorylation of purified His-PKM2 by recombinant active JNK1 paralleled an increase in PK activity in a dose-dependent manner (Fig. 8e), which is consistent with the enhanced activity of PKM2 in PARP14-depleted HCC and JNK1^CA^-transfected HEK293T cells (see Fig. 4a and Fig. 7a). Because knockdown of PARP14 did not affect tyrosine phosphorylation (Tyr105) and acetylation of PKM2 (Fig. 8f,g), two types of post-translational modifications of PKM2 known to inhibit PKM2 (refs 46, 51, 52), these results suggest that active JNK1 stimulates PKM2 by a separate and previously unknown mechanism.

### JNK1 phosphorylates PKM2 at Thr365

**Figure 9:**
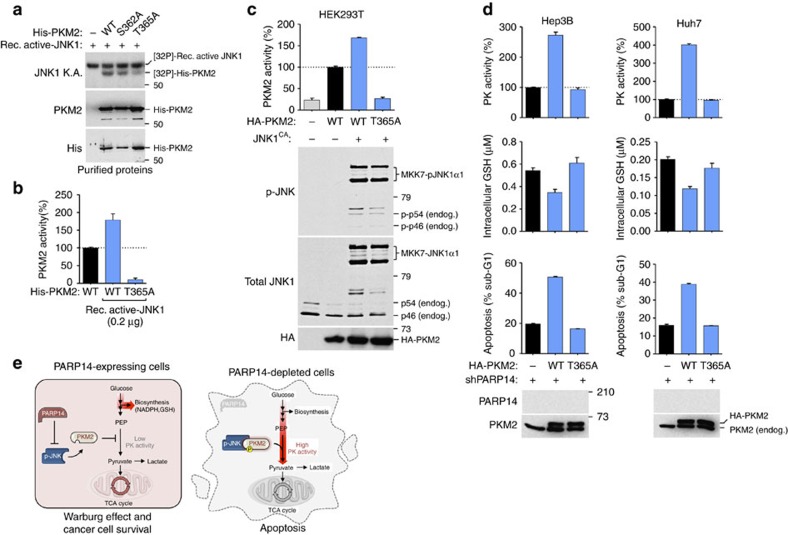
Mutation of Thr365 inhibits PKM2 and promotes PARP14-mediated HCC cell survival. (**a**) *In vitro* JNK1 KA performed by incubating recombinant activated JNK1 (rec. active JNK1) with His-tagged PKM2(WT), PKM2(S362A) or PKM2(T365A) as substrates. (**b**) PKM2 enzymatic activity was assessed incubating purified His-PKM2(WT) or His-PKM2(T365A) in the presence or absence of rec. active JNK1. (**c**) PKM2 enzymatic activity was evaluated in lysates of HEK293T cells transfected with JNK1 constitutive active (JNK1^CA^; 15 μg) or control empty vector (−) in combination with HA-PKM2(WT) or HA-PKM2(T365A) (15 μg). Levels of phosphorylated endogenous (endog.) JNK1 and JNK1^CA^ (MKK7-JNK1α1), and HA and PKM2 were visualized by WBs using appropriate antibodies. Total JNK1 serves as loading control. (**d**) Shown is the PK enzymatic activity, levels of reduced glutathione (GSH) and percentage of apoptotic cells in the sub-G1 (DNA content) population in PARP14-depleted HCC cells expressing empty vector (−), HA-PKM2(WT) or HA-PKM2(T365A). WBs showing the PARP14 knockdown efficiency and ectopic expression of HA-PKM2. (**b**–**d**) Data shown are mean±s.e.m. of three independent experiments. (**e**) Schematic illustration depicting metabolic changes in the presence (left) or absence (right) of PARP14 in HCC cells. The high levels of PARP14 expression in HCC cells halt JNK1-mediated phosphorylation of PKM2, which contribute to maintain low PKM2 activity required for the aerobic glycolysis. The low-active state of PKM2 promotes the Warburg effect and tumour cell survival by diverting glucose metabolites into alternative biosynthetic pathways and sustaining antioxidant responses^3^,^4^,^26^,^41^. Depletion of PARP14, on the other hand, unleashes ‘active JNK1' to phosphorylate PKM2 enhancing its metabolic PK activity necessary for the conversion of glucose in pyruvate, thus lowering antioxidant responses and promoting apoptosis.

In support of PKM2 being a JNK1 substrate, two phosphorylation sites were detected in PKM2, Ser362 and Thr365 by mass spectrometry (Supplementary Fig. 7a,b). Notably, mutation of Thr365, but not Ser362, to alanine completely abolished the JNK1-mediated phosphorylation *in vitro* (Fig. 9a), indicating that Thr365 is a residue phosphorylated by JNK1. Moreover, the recombinant PKM2(T365A) mutant, unlike its wild-type (WT) counterpart, was not activated by active JNK1 *in vitro*, and displayed a markedly lower PK activity than PKM2(WT) even in the absence of JNK1 (Fig. 9b). This suggests that the Thr365 residue might be also critical for the basal enzymatic activity of PKM2. Similar results were observed in HEK293T cells (Fig. 9c). Hence, JNK1 directly binds to and phosphorylates PKM2 at Thr365, which is required for PKM2 activation. Moreover, we performed kinetic analyses of PKM2(WT) and PKM2(T365A) in cells and found that active JNK1 lowered the Michaelis–Menten constant (*K*_m_) of PKM2 for ADP and PEP by 2.1-fold and 1.1-fold, respectively (Supplementary Fig. 8a,b), thus effectively increasing the affinity of PKM2 for these substrates. Last, we examined the biological significance of JNK-mediated PKM2 Thr365 phosphorylation on PARP14 function in HCC cells. Compared with PKM2(WT)-expressing PARP14-depleted HCC cells, PKM2(T365A)-expressing PARP14-depleted HCC cells exhibited reduced levels of PKM2 activity that correlated with a significant increase of GSH levels and decrease in apoptosis (Fig. 9d), supporting a role for PKM2 Thr365 phosphorylation in regulating PARP14-mediated HCC cell survival.

## Discussion

Increased aerobic glycolysis (Warburg effect) is a distinctive feature of rapidly proliferating cells^1^,^2^,^4^. This metabolic signature enables dividing cells to satisfy anabolic and energetic needs for biomass production and to suppress apoptotic signalling^3^,^4^,^26^,^41^. Accordingly, many human cancers including HCC display an aerobic glycolytic phenotype, which often correlates with tumour progression and worse clinical outcomes in cancer patients^5^,^6^,^7^,^8^,^30^,^31^,^32^,^33^. Therefore, understanding how the Warburg effect is regulated in cancer is particularly relevant for identifying new therapeutic interventions. The low enzymatic activity of PKM2 was shown to be a prominent driver of the Warburg effect and cancer cell survival^21^,^25^,^27^,^28^,^46^. Nevertheless, the molecular mechanisms that link the Warburg effect with cell survival control in cancer cells remain largely unclear.

Here we demonstrate that the pro-survival protein PARP14 promotes the Warburg effect in HCC and reveal a molecular mechanism underlying this effect: PARP14 maintains low PKM2 activity via inactivation of the pro-apoptotic protein JNK1, which belongs to the serine/threonine kinase group^20^. Through this regulation, PARP14 sustains the survival of hepatoma cells both *in vitro* and *in vivo* and thereby could be an ideal molecular target in HCC therapy.

Elevated expression levels of PARP14 were found in human HCC cell lines as well as primary tumours, but were absent in normal primary hepatocytes and livers, suggesting a pathogenic role for PARP14 in this disease. Moreover, the expression of PARP14 positively correlated with expression of glycolytic genes in HCC cases, supporting its relevance for the glycolytic phenotype of HCC cells^5^,^6^,^14^,^31^,^32^,^33^. PARP14 levels also appear to have important clinical implications for patients with HCC, as PARP14 expression increases with disease progression from normal livers to cirrhosis and then to active stages of HCC. Remarkably, analysis of cirrhotic livers from patients followed up during a span of 10 years^38^ showed that high PARP14 expression positively correlated with a progression of cirrhosis to HCC and reduced survival rate, suggesting that PARP14 expression levels could be a new biomarker for the risk of HCC. These observations together are consistent with the notion that progression of cirrhosis into HCC is accompanied with a progressive metabolic shift from mitochondrial respiration to aerobic glycolysis that persists into carcinoma^7^,^29^. Given the positive correlation between PARP14 expression and progression of cirrhosis to HCC we speculate that during hepatocarcinogenesis, malignant hepatocytes express PARP14 to rewire their metabolism. Further supporting this idea, PARP14 levels are elevated in early stages, and high PARP14 expression is maintained in late stages of HCC. Interestingly, PARP14 expression is significantly higher in the HpSC-HCC subtype, which has tumour-initiating features with poor prognosis^39^. Establishing the role of PARP14 as a pro-survival factor regulating the Warburg effect, we demonstrate that knockdown of PARP14 impaired the aerobic glycolytic phenotype and survival of both cultured and xenografted hepatoma cells. Thus, although further *in vivo* studies are required, it is reasonable to hypothesize that PARP14 may promote both HCC initiation and maintenance at least in part through upregulation of glycolytic metabolism. Interestingly, knockdown of PARP14 can cooperate with anti-HCC agents in inducing more effective cell death, suggesting that PARP14 could be targeted to improve HCC therapies. Moreover, it is likely that the effects observed in HCC may be generalized to other highly glycolytic cancers as high PARP14 expression was also found in glioblastoma, breast, gastric and lung cancers, and knocking down PARP14 impaired aerobic glycolysis in human cell lines derived from these cancers. Furthermore, in accordance with other studies^17^, the expression levels of PARP14 appear to depend on the levels of the oncoprotein Myc, a key contributor to the Warburg effect in most cancer types^2^,^10^,^11^,^12^.

Recent studies highlight the critical role of the low PK activity of PKM2 in the promotion of the Warburg effect and tumour cell survival by sustaining antioxidant responses^25^,^26^,^27^,^28^,^41^. We indeed found that PARP14 maintains low PKM2 activity in HCC cells. This is demonstrated by the enhanced activity of PKM2 and consequent decrease in NADPH and GSH levels in PARP14-depleted cells that our results show is responsible for the metabolic effects and apoptosis triggered by PARP14 knockdown. So how does PARP14 decrease the activity of PKM2? The enzymatic activity of PKM2 is known to be tightly regulated by the binding of PEP and ADP substrates, allosteric interactions and post-translational modifications^25^,^26^, which include phosphorylation^46^. For instance, tyrosine-phosphorylated peptides bind to PKM2 and decrease its PK activity favouring cell growth^53^. PKM2 itself can be directly phosphorylated by the oncogenic tyrosine kinase FGFR1 at Tyr105, an event associated with low PKM2 activity and diversion of glycolytic intermediates into biosynthetic processes^26^,^46^. The inhibition of PKM2 activity by PARP14 may represent an additional mechanism that modulates PKM2 activity and subsequent aerobic glycolysis in cancer cells. PARP14 restricts PKM2 activity through inactivation of the serine/threonine kinase JNK1, because the enhanced activity of PKM2 in PARP14-depleted cells was reversed by knocking down JNK1 in these cells. Consistent with this, the impaired aerobic glycolytic phenotype and apoptosis caused by PARP14 knockdown in HCC cells were reversed by JNK1 co-silencing, indicating JNK1 as a direct mediator of the metabolic effects and apoptosis triggered by PARP14 knockdown. Thus, in agreement with our previous findings^19^ and the well-known association between glucose metabolism and apoptosis^3^,^9^,^54^,^55^, PARP14 inhibits the pro-apoptotic activity of JNK1 to promote the Warburg effect and consequent survival in HCC cells. Although we cannot rule out that other JNK1-coupled kinases could also be responsible for PKM2 phosphorylation in cells, we show that JNK1 is a PKM2 kinase that directly phosphorylates PKM2 at Thr365 *in vitro* resulting in PKM2 activation, thus expanding the number of kinases that regulates PKM2 activity as well as contact points between apoptosis and glucose metabolism^26^,^41^,^54^,^55^. Moreover, we show that the forced expression of PKM2(T365A) mutant resistant to phosphorylation in PARP14-depleted cells impaired PKM2 activation and reverted the GSH levels and apoptosis. Therefore, the suppression of JNK1-mediated PKM2 Thr365 phosphorylation by PARP14 may represent an unexpected mechanism by which HCC cells acquire a survival advantage. Given that JNK1 activity plays instrumental roles in apoptosis, cell proliferation and metabolism^20^, our results underscore the important role of the PARP14-JNK1-PKM2 regulatory axis in a very broad area of cellular processes.

The obvious question that remains to be addressed relates to the mechanisms of increased PKM2 activity caused by JNK1-mediated Thr365 phosphorylation. PKM2 activity is also regulated by complex structural modifications^28^,^53^. Our kinetic analyses of PKM2(WT) and PKM2(T365A) in cells show that active JNK1 increased affinity of PKM2 for ADP and PEP substrates. In addition, PKM2 can translocate to the nucleus, where it serves as a transcriptional co-activator to induce HIF1α-dependent glycolytic gene expression^24^. Furthermore, phosphorylation of PKM2 at Ser37 results in the nuclear translocation of PKM2 and upregulation of Myc glycolytic target genes^48^. Interestingly, we found that, compared with control cells, cells depleted of PARP14 exhibit reduced levels of nuclear PKM2 and impaired transcriptional activity of HIF1α and Myc with a corresponding decrease in expression levels of glycolytic enzymes, suggesting a role for PARP14 in the nuclear function of PKM2. Therefore, it is possible that JNK1-mediated Thr365 phosphorylation may directly cause conformational changes in ADP- and PEP-binding site and/or modulate the nuclear translocation/function of PKM2. Future investigations are warranted to address this question.

In summary, our findings delineate an unexpected pathway regulating the Warburg effect required for HCC cell survival whereby PKM2 activity is negatively regulated by the PARP14-JNK1 axis (Fig. 9e), thus constituting an additional paradigm of how cell metabolism and evasion of apoptosis are inextricably linked^3^,^9^,^54^,^55^. These results may lead to design new therapeutic strategies for human HCCs.

## Methods

### Gene expression profiling and immunohistochemistry

Gene expression profiling studies involving multiple clinical samples were performed analysing the expression of specific transcripts in different data sets available through Gene Expression Omnibus. Formalin-fixed paraffin-embedded HCC and cirrhotic specimens used in tissue microarrays were commercially obtained from US Biomax (TMA no. BC03117). All human tissues were collected in accordance with the Anatomical Gift Act as for Sample Collection Policy by US Biomax. Immunohistochemical staining of paraffin sections was carried out using a two-step protocol. After antigen retrieval, the slices were incubated with anti-human PARP14 antibody (HPA012063, Sigma-Aldrich; 1:50). According to the intensity and total area of the staining, the expression of PARP14 was scored as either strong expression (expressed >50% of cells) or low expression (<50% of cells). A qualified liver pathologist (R.A.A.) performed the immunohistochemical scoring without knowledge of samples identity.

### Antibodies and reagents

The antibodies used for WBs were as follows: PARP14 (HPA012063, Sigma-Aldrich, 1:2,000), α-actinin (sc-7454R, 1:1,500) and β-actin (sc-1616R; 1:1,000; Santa Cruz Biotechnology); phospho-JNK (#9251), phospho-PKM2(Tyr105) (#3827), pan-K-acetylated (#9814), HK2 (#2867), PFK (#8164), LDHA (#3582), PDK1 (#3205), Myc (#5605), GAPDH (#5174; Cell Signaling, 1:1,000); JNK1 (BD-51-1570R, BD Bioscience; 1:1,000); PARP1 (AM30, Calbiochem; 1:1,000); Caspase-3 (#9665, Cell Signaling; 1:800); PKM2 (#3827, Cell Signaling; 1:1,000 for WB; 1:50 for IP); PKM1/2 (#3190, Cell Signaling, 1:1,500); PKM1 (SAB4200094, Sigma; 1:1,000); HA-probe (sc-2362, Santa Cruz Biotechnology; 1:800); FLAG-probe (F1804, Sigma-Aldrich; 1:2,000); His-probe (#34698, Qiagen; 1:2,000); Histone-H3 (Active motif; 1:3,000); donkey anti-rabbit HRP conjugated (NA9340V; GE Healthcare; 1:3,000); and goat anti-mouse HRP conjugated (sc-2031, Santa Cruz Biotechnology; 1:1,000). The antibodies used for IP were as follows: FLAG M2-affinity gel (A2220, Sigma-Aldrich; 20 μl per reaction) and JNK1 (0.5 μg per reaction).

The following reagents were purchased from Sigma-Aldrich: PK from rabbit muscle, crystal violet, Coomassie blue, propidium iodide, oligomycin, carbonyl cyanide 4-(trifluoromethoxy) phenylhydrazone, antimycin A, 2-deoxyglucose, PJ-34 and Myc-inhibitor 10058-F4. Sorafenib (BAY43-9006) and doxorubicin were purchased from Enzo Life Sciences and Abcam, respectively.

### Cell culture and shRNA lentiviral infections

Human SK-Hep-1, Snu-449 and MCF7 cell lines were obtained from ATCC. Hep3B, Huh7, PLC5 and HepG2 cell lines were kindly supplied by H. Walczak and described elsewhere^56^,^57^. RPMI-8226 cell line is from our lab stock and previously described^19^. Human U87 glioma cells were generously provided by P. Salomoni (UCL Cancer Institute). Huh7, Hep3B, HepG2, PLC5, MCF7 and U87 cells were cultured in Dulbecco's modified Eagle's medium (DMEM, 25 mM glucose; Life Technologies); Snu-449 and RPMI-8226 were cultured in RPMI1640 medium (11.11 mM glucose; Life Technologies). Nontumoural IHHs were maintained in DMEM-F12 as described^36^,^37^. All mediums were supplemented with 10% fetal bovine serum, antibiotics (100 U ml^−l^ penicillin and 100 mg ml^−l^ streptomycin) and 2 mM glutamine (Life Technologies). Cells were maintained at 37 °C in a humidified cell incubator containing 20% O_2_, 5% CO_2_ in air (referred as normoxia). For hypoxia treatments, cultured cells were sealed in a humidified modular incubator chamber (Billupus-Rothemberg Inc.), flushed with a gas mixture of 1% O_2_, 5% CO_2_ and 94% N_2_ for 15 min and incubated at 37 °C for the time indicated. Transient transfections of DNA plasmids, production of high-titer lentiviral preparations in HEK293T cells and lentiviral infections were carried out as described previously^19^.

### Expression plasmids and mutagenesis

Expression plasmids for shRNAs were made in a pLL3.7 vector^19^. The targeted sequences were: human *PARP14*, 5′-GGAAAGGGCTCACTCACAA-3′ (ref. 19); human *PARP14*#2, 5′-GAAAGCATGTGTATTATGT-3′; human *PKM2*, 5′-ctgtggctctagacactaa-3′; and human *JNK1*, 5′-GGAGCTCATGGATGCAAAT-3′ ref. 19. Expression plasmids for HA-JNK1 and HA-JNK2 were previously described^19^. Expression plasmid of JNK1 constitutive active (JNK1^CA^; LZRS-FLAG-MKK7-JNK1a1) was a gift from J. Zhang (Duke University)^50^. The full-length complementary DNAs of human PKM2 and PKM1 were obtained from pWZL-FLAG-PKM2 (Addgene plasmid 20585)^58^ and pET28-hPKM1 (Addgene plasmid 44241)^28^, respectively, and then cloned between BamHI and XhoI sites of either pcDNA-HA- or pcDNA-FLAG-expressing vectors. pcDNA-HA-PKM2 mutant forms (S362A and T365A) were generated using the QuickChange XL site-directed mutagenesis kit (Stratagene). FLAG-PKM1, HA-PKM2 WT and T365A were then cloned in pWPI lentiviral vector between PmeI site^59^. For bacterial expression, PKM1, PKM2(WT) and PKM2 mutants were cloned between BamHI and XhoI sites of pET28b vector (Novagen). pGEX-c-Jun was previously described^19^.

### Soft agar colony assays

Hep3B and HepG2 cells expressing nonspecific (shNS) or PARP14 (shPARP14) shRNAs were collected at day 4 post-infection and mixed at a density of 3 × 10^4^ cells with 0.6% low melting point agarose (Sigma-Aldrich) in complete DMEM phenol red-free medium containing growth medium for a final concentration of 0.3% agarose. The cell mixture was plated on top of a solidified layer of 0.6% agarose in growth medium. Cells were fed every 3–4 days with growth medium. The number of colonies was automatically counted at the indicated time using the open-source software OpenCFU^60^.

### *In vivo* xenograft tumour model

Exponentially growing Huh7 cells expressing nonspecific (shNS) or PARP14 (shPARP14) shRNAs were collected, washed and resuspended in sterile PBS. Equal numbers (2.3 × 10^6^) of Huh7-shNS or Huh7-shPARP14 cells were inoculated subcutaneously into the left and right flanks of 6-week-old female NOD/SCID (Charles River) immunodeficient mice, respectively. Tumour growth was monitored twice a week and mice were killed at the time indicated when the tumour was early detectable (∼1 cm) on either flank. Tumours were removed, weighed and photographed. Tumour volume was determined using the formula: *L* × *W*^2^ × 0.5, where *L* is the longest diameter and *W* is the shortest diameter of the excised tumour. All experiments were carried out with UK Home Office Authority and Imperial College Ethical Review Process Committee approval (PPL 70/6874).

### Cell-based assays and microscopy analyses

For viability and death assays, cells were seeded onto six-multiwell plates and collected at the time indicated in each experiment. Cell number was determined by manual counting of adherent cells. Viability assay was assessed using alamarBlue (Life Technologies) according to the manufacturer's protocol. Apoptosis was detected using propidium iodide nuclear staining of pooled, detached and adherent cells as previously reported^19^. Light microscopy images were acquired using either an Optika XDS microscope with a 10 × objective adapted with a 3.0-MP camera (Moticam). For electron microscopy, Hep3B cells expressing nonspecific (shNS) or PARP14 (shPARP14) shRNAs were fixed with 2% paraformaldehyde, 1.5% glutaraldehyde in cacodylate buffer (0.1 M, pH 7.3). Cells were post-fixed in 1% aqueous osmium tetroxide (OsO_4_) and 1.5% potassium ferrocyanide (Sigma-Aldrich) in cacodylate buffer, dehydrated in a graded ethanol–water series, cleared in propylene oxide and infiltrated with Agar 100 resin. Ultrathin sections (70 nm) were cut using a diamond knife on a Reichert ultramicrotome, collected on 300 Ni mesh grids and stained with uranyl acetate and lead citrate. Cells were observed in a Jeol 1010 transition electron microscope (Jeol USA Inc.) and the images were recorded using an Orius CDD camera (Gatan Inc).

### Analysis of glucose metabolism and mitochondrial activity

Cells were seeded onto 35-mm culture dishes, and after 6 h the culture medium was replaced with fresh complete medium and incubated for additional 48 h. The media were then collected for measurement of glucose and lactate concentration and cells harvested for protein lysates. Glucose levels were determined using a glucose assay kit (Sigma-Aldrich). Glucose consumption was calculated by deducting the measured glucose concentration in the media from the original glucose concentration. Lactate levels were determined using a lactate assay kit (Trinity Biotech) according to the manufacturer's instruction. All values were normalized on the basis of the Bradford protein assay. *In vivo* cells real-time ECAR and OCR were monitored with the Seahorse XF24 Flux Analyser (Seahorse Bioscience), according to the manufacturer's instructions. Cells (37,500 or 50,000) were seeded in a XF24-well plate containing complete medium. For assessment of the real-time glycolytic rate (ECAR), cells were incubated with unbuffered media followed by a sequential injection of 10 mM glucose, 1 μM (Huh7) or 2 μM (Hep3B) oligomycin and 80 mM 2-deoxyglucose. The mitochondrial respiration (OCR) was assessed using sequential injection of 1 μM (Huh7) or 2 μM (Hep3B) oligomycin, 0.2 μM carbonyl cyanide 4-(trifluoromethoxy) phenylhydrazone and 2 μM antimycin A. Both ECAR and OCR measurements were normalized to cell number. Activity of mitochondrial respiratory chain complex I in cellular lysates was assessed using Complex I Enzyme activity microplate assay kit (Abcam) according to the manufacturer's protocol. All values were normalized on the basis of the Bradford protein assay.

### Quantification of glycolytic intermediates

Levels of pyruvate, reduced GSH, in lysates of HCC cells were analysed using Pyruvate Assay Kit (Abcam) and GSH-Glo glutathione assay (Promega), respectively, according to the manufacturer's protocol. NADPH levels were determined using the NADP+/NADPH quantification kit (Biovision). Intracellular ATP levels were determined in cell lysates using the Luminescence ATP Detection Assay System (Perkin-Elmer) according to the manufacturer's protocol. Levels of glucose-6-phosphate and 2-phosphoglycerate were analysed using Glucose-6-Phosphate (Biovision) and 2-Phosphoglycerate (Abcam) Assay Kit, respectively, according to the manufacturer's protocol. All values were normalized on the basis of the Bradford protein assay.

### Western blot and co-immunoprecipitation

Tumour cells or xenografted tumour tissues were homogenized in modified lysis buffer (50 mM Tris-HCl pH 7.5, 100 mM NaCI, 50 mM NaF, 1 mM Na_3_VO_4_, 30 mM sodium pyrophosphate, 0.5% NP-40 and 0.5 mM PMSF (Sigma-Aldrich) supplemented with EDTA-free protease inhibitor cocktail (Roche)). Healthy and HCC human liver lysates were commercially obtained from OriGene Technologies. Primary human hepatocytes from normal livers were obtained from Life Technologies. Once thawed, cryopreserved hepatocytes were lysed in modified lysis buffer and processed for WBs analyses. WB and co-IP were performed as described previously^19^. Briefly, for IP, lysates were incubated with either 20 μl of FLAG M2-affinity gel (Sigma-Aldrich or 30 μl of a 1:1 slurry of protein A/G Plus-agarose (Santa Cruz Biotechnology) in presence of a specific antibody for at least 4 h at 4 °C. The beads were washed four times with 1 ml of lysis buffer and then subjected to SDS–polyacrylamide gel electrophoresis analysis. Where indicated densitometry analyses were performed using Image J analysis software (National Institutes of Health, USA), as previously described^19^. Uncropped scans of WBs are provided in Supplementary Fig. 9.

### Kinase assays

JNK1 KAs were performed as described previously^19^. Briefly, *in vitro* JNK1 KA was performed using purified glutathione S-transferase c-Jun(1–79), His-PKM2 WT or mutants forms (S362A and T365A) as substrates. The *in vitro* phosphorylation was performed in the presence of [γ^32^P]ATP (Perkin-Elmer) for 40 min at 30 °C. Reactions were stopped by the addition of SDS sample buffer, boiled and the phosphorylated proteins were resolved by 10% SDS–polyacrylamide gel electrophoresis. The gel was dried and subjected to radiography.

### Subcellular fractionation and transcriptional activity

Fractioning of nuclear and cytosolic extracts was performed using NE-PER nuclear and cytoplasmic extraction kit (Thermo Scientific) according to the manufacturer's protocol. Transcriptional activity of Myc and HIF1α was analysed using TransAm Myc and TransAm HIF1 Activation Assay (Active Motif) according to the manufacturer's protocol. Briefly, for Myc activity, lysates were prepared from nuclear and cytoplasmic fractions of shPARP14- and shNS-expressing Hep3B cells cultured in normal standard conditions. For HIF1α, nuclear lysates were prepared from shPARP14- and shNS-expressing Hep3B cells cultured in normal standard conditions (normoxia) and in hypoxic conditions for 12 h (hypoxia).

### Pyruvate kinase enzymatic activity

PK activity in cells was measured by monitoring the conversion of pyruvate to lactate coupled with the conversion of NADH to NAD+ by the lactate dehydrogenase, as reported^28^. Briefly, the assay was carried out using an optimal concentration of cell lysates combined with a 1 × PK reaction buffer (50 mM Tris-HCl pH 7.5, 100 mM KCl and 5 mM MgCl2 containing 0.5 mM PEP (Sigma), 0.6 mM ADP (Sigma), 660 μM NADH (Sigma) and 8 units lactate dehydrogenase (Sigma)). The final reaction volume was 100 μl in 96-well plates. The decrease in absorbance at 340 nm from the oxidation of NADH was measured as PK activity by a FLUOstar Omega spectofotometer (BMGH Labtech). PK activity of purified proteins was measured using 0.15–2 μg of recombinant human His-PKM2(WT) or His-PKM2(T365A) pre-incubated with recombinant active JNK1 for 15 min at room temperature. The mixture was then assessed with 1 × PK reaction buffer. To cross-validate the results, in most cases, the PK activity was also assessed using an alternative PK Assay method (Biovision) according to the manufacturer's protocol.

### Purification of recombinant proteins

Recombinant active JNK1 was purchased from Abcam. pET28-PKM1, pET28-PKM2(WT) and mutants forms (S362A and T365A) were expressed in bacteria and purified as previously reported^61^. Briefly, transformed *Escherichia coli* BL21(DE3) cells were grown in LB broth containing 2 mM MgCl_2_ and 0.05 mg ml^−l^ kanamycin at 37 °C and induced with 0.5 mM isopropyl-β-D-thiogalactoside for 6 h. Recombinant His-tagged proteins were purified using the Ni-NTA spin columns (Qiagen) according to manufacturer's protocol. Purified recombinant proteins were dialysed against 0.1 M phosphate buffer (pH 7.4) and visualized with Coomassie blue staining method^61^.

### Phosphorylation of PKM2 and mass spectrometry

*In vitro* phosphorylation of PKM2 was performed incubating purified active JNK1 (0.5 μg per reaction) with recombinant His-PKM2 for 40 min at 30 °C in kinase buffer (20 mM HEPES pH 7.6, 20 mM MgCl_2_, 20 mM β-glycerophosphate disodium salt, 2 mM dithiothreitol, 0.1 mM Na_3_VO_4_ and 50 μM cold ATP). Proteins were resolved on a 8% polyacrylamide gel and stained with Coomassie brilliant blue (R250) and subjected to mass spectrometry, as previously reported^62^. Briefly, stained bands corresponding to protein of molecular weight 58 kDa were excised, cut into small (<1 mm^3^) pieces and washed three times by repetitive dehydration and hydration using, respectively, 100% acetonitrile (MeCN) and 100 mM ammonium bicarbonate (Ambic). Proteins were in-gel reduced in the presence of 10 mM dithiothreitol for 1 h at 56 °C and immediately alkylated using 55 mM iodoacetamide, and digested overnight at 37 °C with 100 ng trypsin. Digested peptides were recovered, dried and resuspended in Ambic/0.1% formic acid. Twenty per cent of the peptide mixture was analysed by nano-liquid chromatography–tandem mass spectrometry using an LTQ Velos-Orbitrap MS (Thermo Scientific) coupled with an Ultimate RSLCnano-LC system (Dionex). Briefly, RawMSdata files were processed using Proteome Discoverer v.1.3 (Thermo Scientific). Processed files were searched against the SwissProt human database using the Mascot search engine version 2.3.0. Using a reversed decoy database, false discovery rate was <1%. Searches were done with tryptic specificity allowing up to one miscleavage and a tolerance on mass measurement of 10 p.p.m. in mass spectrometry mode and 0.6 Da for tandem mass spectrometry ions. Structure modifications allowed were oxidized methionine, deamidation of asparagine and glutamine residues and phosphorylated serine, threonine and tyrosine residues all of which were searched as variable modifications. Carbamidomethylated cysteine residues were searched as a fixed modification.

### Statistical analysis

Statistical analyses were performed with GraphPad Prism (Graphpad Software Inc). Unpaired Student's *t*-test (two tailed) and Mann–Whitney test were performed between two groups and one-way analysis of variance followed by Bonferroni's multiple comparison tests were used for statistical comparison between three or more groups. Data in graphs are shown as mean±s.e.m.

## Additional information

**How to cite this article:** Iansante, V. *et al*. PARP14 promotes the Warburg effect in hepatocellular carcinoma by inhibiting JNK1-dependent PKM2 phosphorylation and activation. *Nat. Commun.* 6:78882 doi: 10.1038/ncomms8882 (2015).

## Supplementary Material

Supplementary Information Supplementary Figures 1-9
