# Supplementary material for: PARP14 promotes the Warburg effect in hepatocellular carcinoma by inhibiting JNK1-dependent PKM2 phosphorylation and activation
Source: Nat Commun. 2015 Aug 10;6:7882. doi: 10.1038/ncomms8882 (PMC4918319; doi:10.1038/ncomms8882)

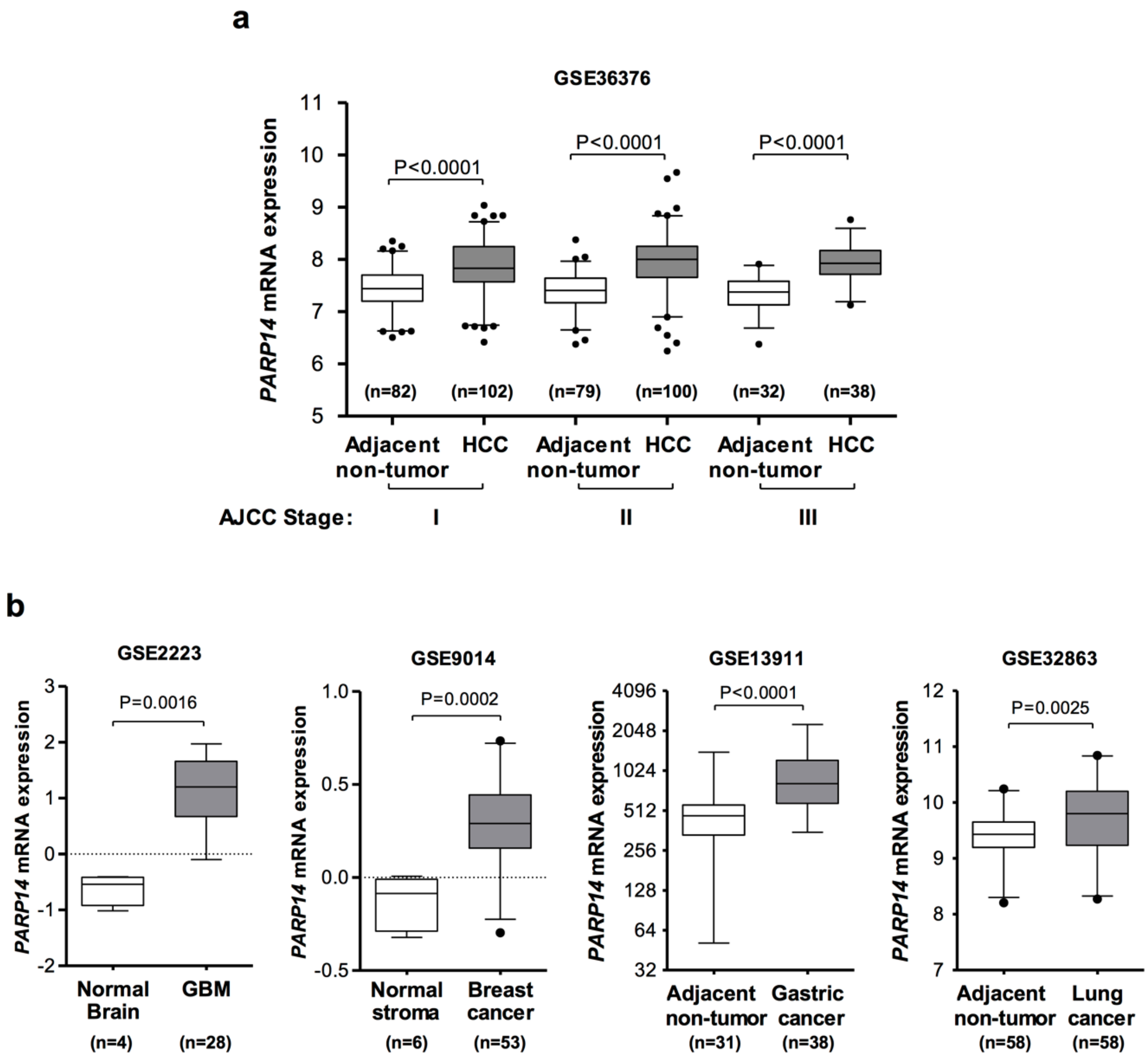

Supplementary Fig. 1. PARP14 is highly expressed in human cancers. (a) Levels of PARP14 transcripts were evaluated in the same microarray dataset as Fig. 1b but classified by AJCC stage. Increased PARP14 expression is observed in early stages compared to adjacent non-tumor tissue and, high PARP14 is maintained in late stages of HCC. (**b**) Gene-expression analysis of PARP14 transcripts in brain (glioma; GBM), breast, gastric and lung cancer patients from the NCBI GEO database showing significantly higher expression of PARP14 in tumor tissues compared to normal or adjacent non-tumor matched tissues. (**a,b**) For each box plot, the whiskers represent the 2.5-97.5th percentile range of values, the lower and up boundaries denote the 25th and 75th percentile of each data set, respectively, and the horizontal line represents the median value for each group. P values were calculated by nonparametric Mann-Whitney tests. n=number of samples for each dataset.

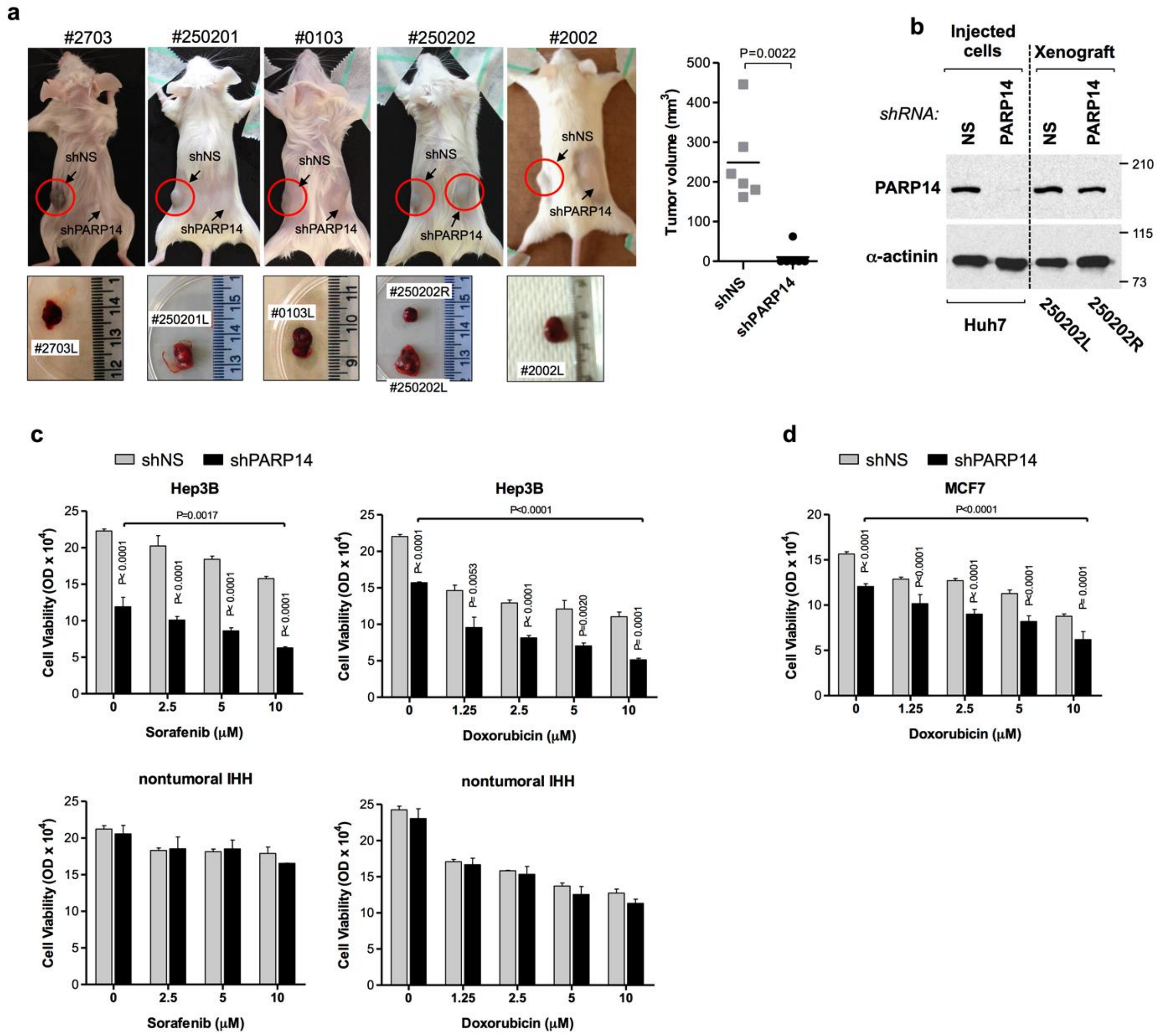

Supplementary Fig. 2. PARP14 is required for tumor growth and modulates tumor cells chemosensitivity. (**a**) Xenograft assay of Huh7 cells expressing either PARP14 (shPARP14) or non-specific (shNS) shRNAs injected into right (R; shPARP14) and left (L; shNS) flanks. Tumor weight was analyzed in excised tumors; the horizontal lines denote the mean of n=6 mice. (**b**) WBs analyses showing PARP14 protein levels in lysates from Huh7 cells stable-expressing shNS or shPARP14 used in the xenograft experiments (Injected cells) and from the explanted tumor (Xenograft). Note that only the mouse #250202 developed a tumor in the right side and, this was smaller in size and had regained PARP14 expression. (**c**) Hep3B and nontumoral IHH cells were infected with the indicated shRNA and treated (at day 1 of infection) with increasing concentrations of Sorafenib (top) or Doxorubicin (bottom). (**d**) MCF7 cells were infected with shPARP14 and control (shNS) shRNAs and treated (at day 1 of infection) with increasing concentrations of Doxorubicin. Cell viability was measured by alamarBlue® assay at 36h (Sorafenib) and 24h (Doxorubicin) after treatment. (**c,d**) Data shown are mean±SEM of three independent cultures. P values were calculated by Student's *t*-test.

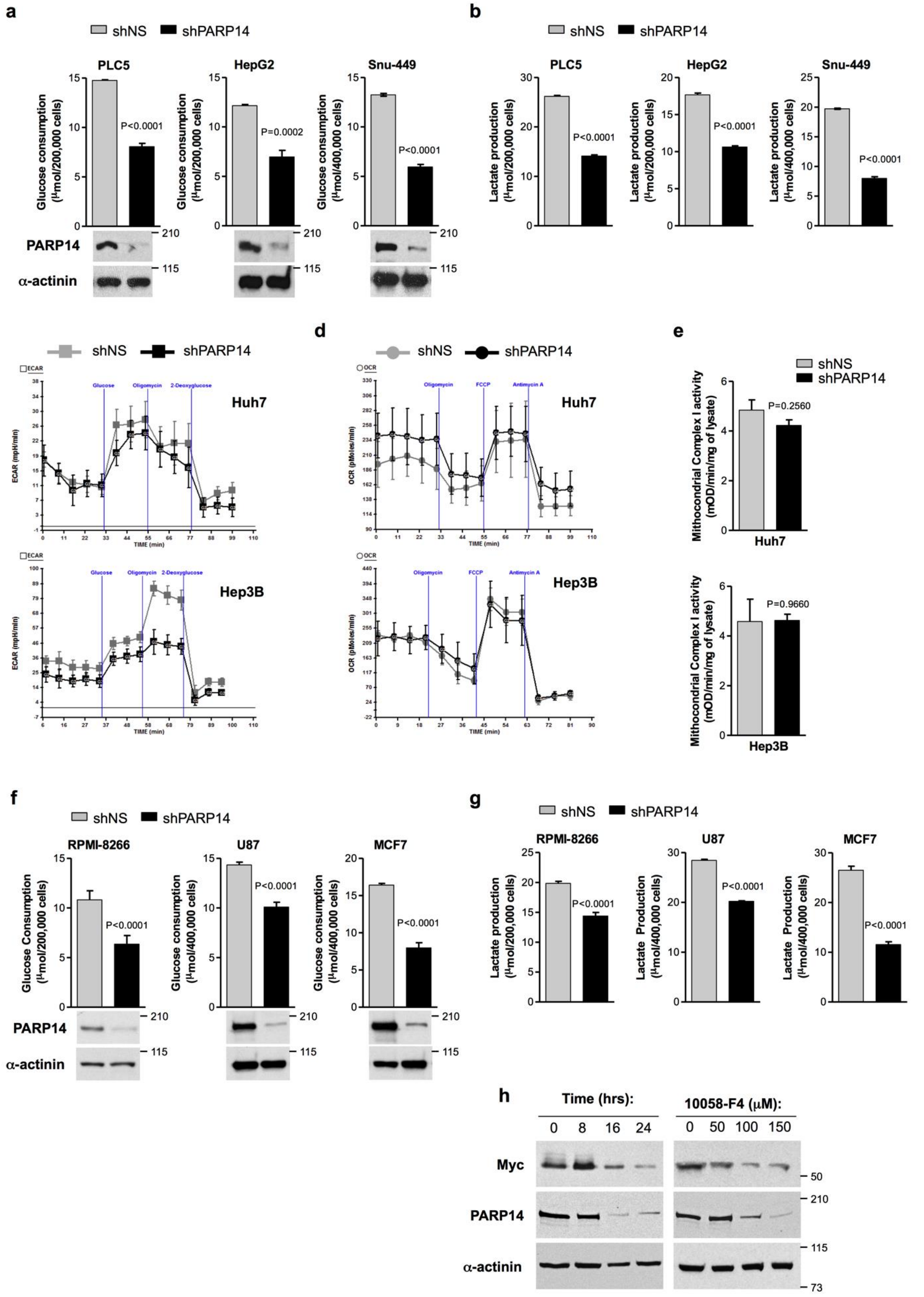

Supplementary Fig. 3. PARP14 is critical for the Warburg effect in human cancers. (**a, b**) Glucose consumption (**a**) and lactate production (**b**) in multiple HCC cells stable-expressing PARP14 or control NS shRNAs (day 4 after shRNA expression). WBs showing levels of PARP14 (knockdown efficiency) in lysates of matching cells. (**c**) Measurements of extracellular acidification rate (ECAR) were obtained before and after sequential injection of glucose (initiates glycolysis), oligomycin (eliminates mitochondrial ATP production) and 2-deoxyglucose (inhibits glycolysis).

Raw data of a typical experiment in Huh7 and Hep3B cells expressing shPARP14 or shNS are shown. Values indicate the mean±SD of five independent cultures. (**d**) Measurement of oxygen consumption rate (OCR) in response to sequential injection of oligomycin (eliminates mitochondrial ATP production), FCCP (protonophore uncoupler) and antimycin A (inhibits the electron transport chain) in HCC cells expressing shPARP14 or shNS. (**e**) Bar graph showing the activity of mitochondrial respiratory chain complex I in control and shPARP14 Huh7 and Hep3B cells. (**f, g**) Glucose consumption (**f**) and lactate production (**g**) in a variety of cancer cell lines (RPMI-8266 multiple myeloma, U87 glioblastoma, MCF7 breast carcinoma) expressing either PARP14 or control NS shRNAs. WBs showing levels of PARP14 (knockdown efficiency) in lysates of matching cells. Values indicate the mean±SEM of three independent cultures. (**h**) WBs showing levels of PARP14 in HepG2 cells after treatment with 100μM 10058-F4 at the indicated time (left) or after 48 hours treatment with the different concentration of 10058-F4 (right). (**a,b,f,d**) Data shown are mean±SEM of n≥3 technical replicates and are representative of at least three independent experiments. P values were calculated by Student's *t*-test. (**c,d**) Data shown are mean±SD of five independent cultures. P values were calculated by Student's *t*-test.

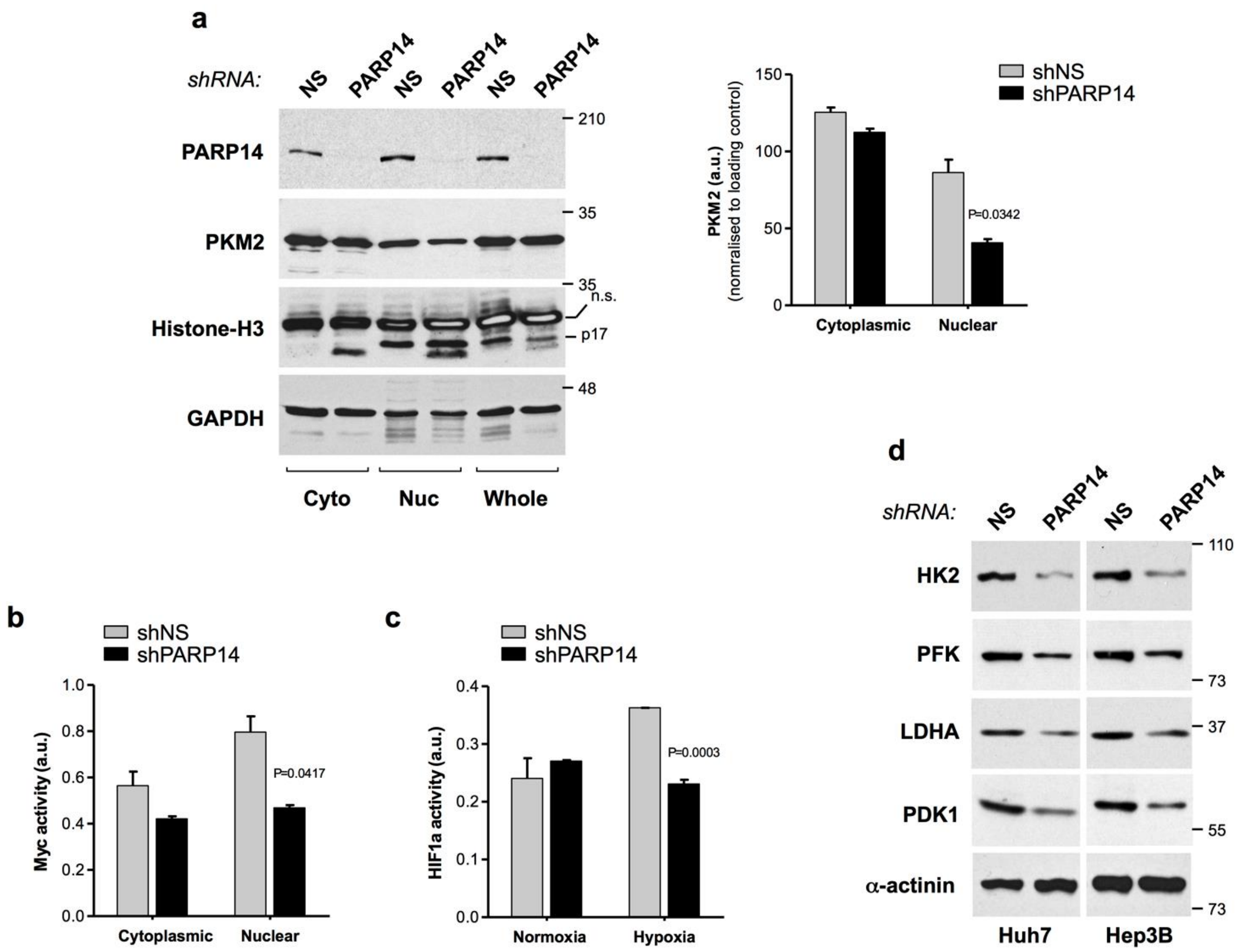

Supplementary Fig. 4. PARP14 affects the nuclear function of PKM2. (**a**) WBs showing protein levels of PARP14 and PKM2 in nuclear (Nuc), cytoplasmic (Cyto) and whole cell lysates of shPARP14-expressing Hep3B cells compared to control shNS-expressing cells. Histone-H3 and GAPDH were used as loading control for nuclear and cytoplasmic fractions, respectively. Data in the graph shown the densitometry analyses of n=2 different blots. P values were calculated by Student's *t*-test. (**b,c**) Transcriptional activity of Myc (c) and HIF1α (d) in shPARP14-expressing Hep3B cells compared to control shNS-expressing cells cultured either in normal standard conditions (normoxia) and hypoxic conditions for 12 hours (hypoxia). a.u. arbitrary units. (**d**) WBs showing protein levels of glycolytic enzymes hexokinase II (HK2), phosphofructokinase (PFK), lactate dehydrogenase A (LDHA), pyruvate dehydrogenase kinase (PDK1) in shPARP14-expressing Huh7 and Hep3B cells compared to control shNS-expressing cells. Data shown are mean±SEM of three independent experiments. P values were calculated by Student's *t*-test.

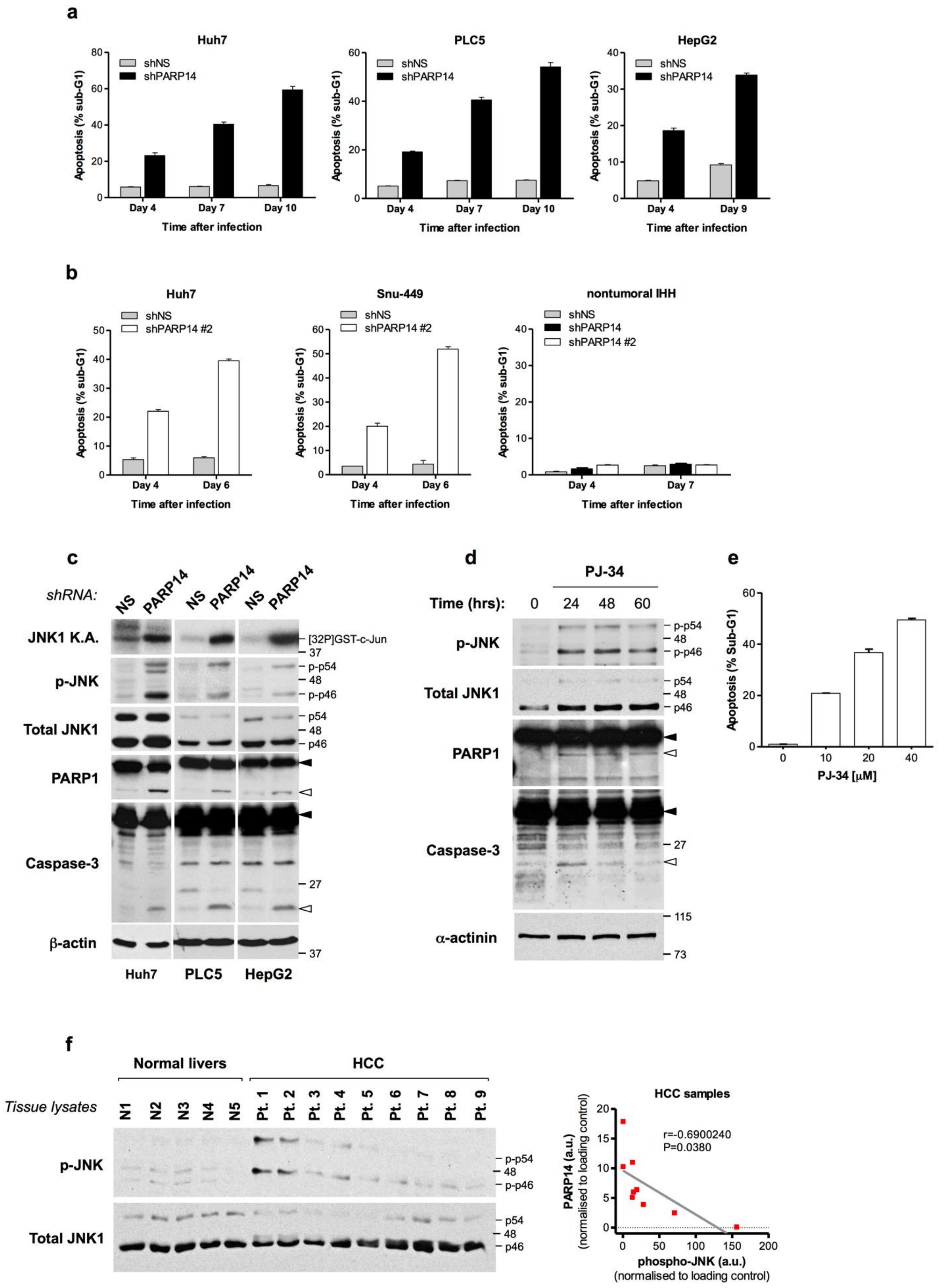

Supplementary Fig. 5. PARP14 is required for HCC cell survival. (**a, b**) Percentage of apoptosis in the population sub-G1 (DNA content) of multiple HCC and nontumoral IHH cells expressing shNS, shPARP14 or shPARP14#2. (**c**) WBs analyses of lysates from multiple HCC cell lines expressing shNS or shPARP14 showing cleavage of caspase-3 and PARP1 and phosphorylation of JNK (p-JNK). Closed and open arrowheads indicate the pro-cleaved and cleaved (active) products of the indicated proteins, respectively. p54 and p46 denotes the JNK

splicing isoforms. *In vitro* JNK1 kinase activity (JNK1 K.A.) was performed using GST-c-Jun as substrate. (**d**) WBs analyses of lysates from Hep3B cells left untreated or treated with 20μM PJ-34 at different time showing cleavage of caspase-3 and PARP1 and p-JNK. (**e**) Apoptosis of Hep3B cells left untreated or treated with PJ-34 in a dose-dependent manner for 48 hours. (**f**) Left, WBs analyses showing p-JNK in HCC tissues lysates used in Figure 1d. Right, scatterplot showing the negative correlation between PARP14 and p-JNK expression in HCC tissue lysates. Densitometry analysis was performed to assess the levels of p-JNK (normalized to total JNK1 levels) and PARP14 (normalized to α-actinin levels) (see also Figure 1d). Pearson's coefficient tests were performed to assess statistical significance. a.u. arbitrary units. (**a,b,e**) Data shown are mean±SEM of three independent cultures. P values were calculated by Student's *t*-test.

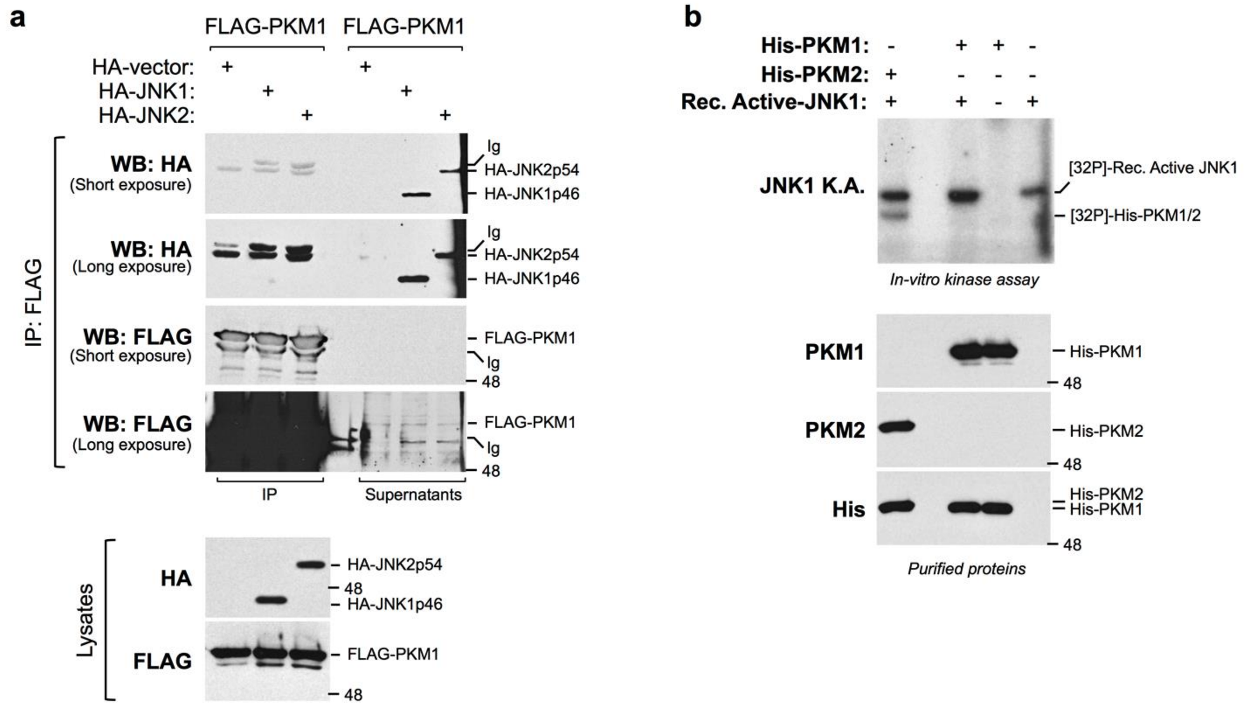

Supplementary Fig. 6. PKM1 is not a substrate of JNK1. (**a**) Immunoprecipitation (IP) analyses in HEK293T cells transfected with FLAG-PKM1 in combination with HA-JNK1, HA-JNK2 or empty vector. Levels of FLAG-PKM1 in supernatants (inputs after IP) are shown to visualize the complete immunoprecipitation of FLAG-PKM1 from lysates. (**b**) *In-vitro* kinase assay was performed by incubating recombinant activated-JNK1 (Rec.Active-JNK1) with His-PKM1 or His-PKM2 (positive control). [32P]-Rec.Active-JNK1 denotes the auto-phosphorylated protein. WBs of the recombinant proteins used in the assay showing the specificity and purity of the purified proteins.

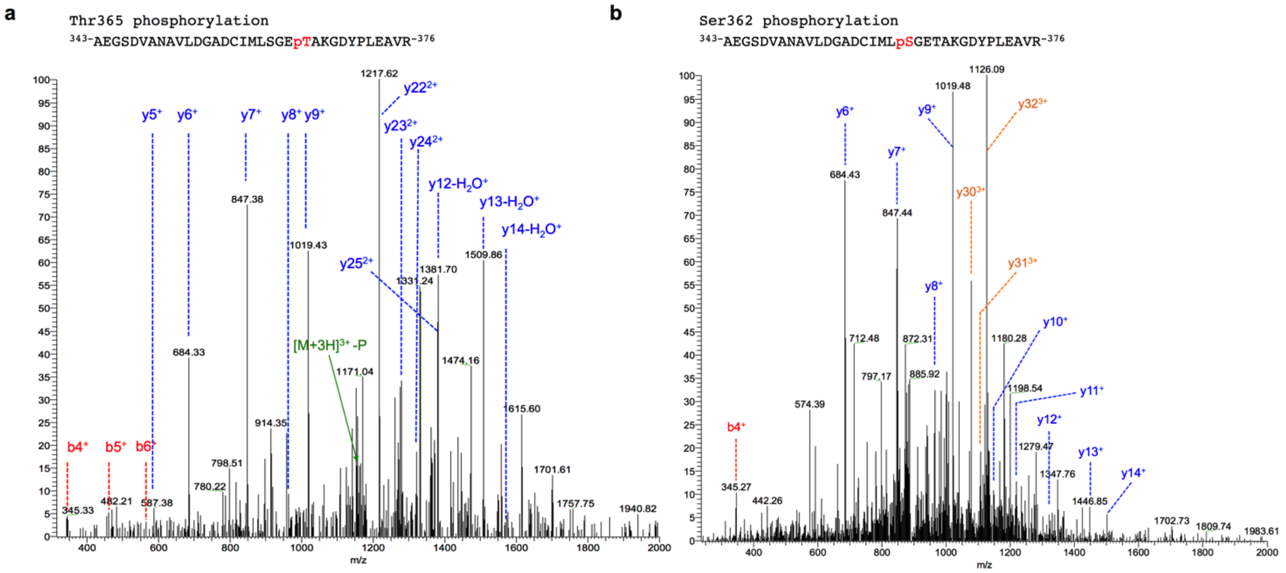

Supplementary Fig. 7. JNK1 phosphorylates PKM2 at Thr365. (**a, b**) His-PKM2 proteins phosphorylated by active JNK1 *in vitro* were triptic digested and then analyzed by mass spectrometry. (**a**) MS analyses of a tryptic fragment at m/z 1192.21436 matched to the triply charged $[M+3H]^{3+}$ peptide $^{343-}$AEGSDVANAVLDGADCIMLSGETAKGDYPLEAVR^{-376}, suggesting that Thr365 was phosphorylated. Relevant y-ions (blue) and b-ions (red) are shown. The presence of $b4^{+}$, $b5^{+}$ and $b6^{+}$ ions indicates that Ser346 is unmodified; the presence of $y12–H_2O^{+}$ to $y14–H_2O^{+}$ ions is in agreement with the assignment of the phosphorylation site to Thr365. The presence of a phosphate at Thr365 is also confirmed by the identification of the precursor ion $[M+3H]^{3+}$ at m/z 1160.04 minus the phosphate group (green). The peptide was identified with a Mascot score of 41, expectation value of 0.0063 and Δm of 5.76ppm. (**b**) MS analyses of a tryptic fragment at m/z 894.6538 matched to the quadruply charged $[M+4H]^{4+}$ peptide $^{343-}$AEGSDVANAVLDGADCIMLSGETAKGDYPLEAVR^{-376}, suggesting that Ser362 was phosphorylated. Relevant y-ions (blue), b-ions (red) and the triply charged fragment ions (orange) are shown. The presence of the $b4^{+}$ ion and y-ion series $y6^{+}$-$y14^{+}$, respectively, indicates that Ser346 and Thr365 are unmodified; the presence of the triply charged $y30^{3}$, $y31^{3+}$, $y32^{3+}$ ions suggests Ser362 as the phosphorylation site. The peptide was identified with a Mascot score of 14, expectation value of 2.8 and Δm of 0.48ppm.

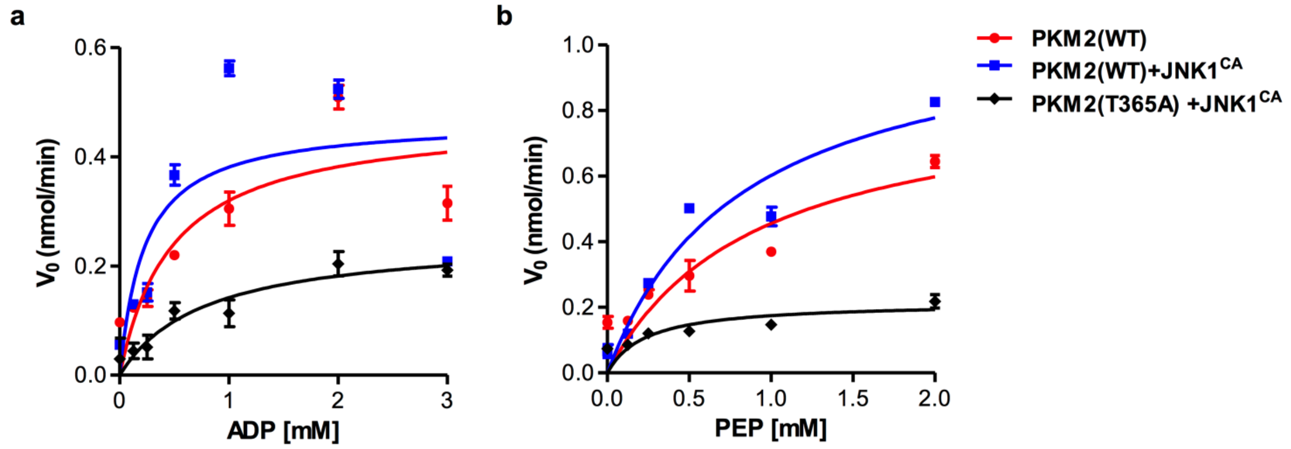

Supplementary Fig. 8. JNK1-mediated PKM2 Thr365 phosphorylation enhances PKM2 activity. (**a, b**) PKM2 enzymatic activity was evaluated with increasing concentrations of ADP (**a**) or PEP (**b**) in lysates of HEK293T cells transfected with JNK1 constitutive-active ($JNK1^{CA}$) or control empty vector in combination with HA-PKM2(WT) or HA-PKM2(T365A). K_m values for ADP were determined as 0.4835±0.1950 mM, 0.2264±0.1367 mM, 0.7798±0.3425 mM in lysates containing PKM2(WT), PKM2(WT)+$JNK1^{CA}$, PKM2(T365A)+$JNK1^{CA}$, respectively. K_m values for PEP were determined as 0.9096±0.3780 mM, 0.8242±0.2204 mM, 0.2345±0.1128 mM in lysates containing PKM2(WT), PKM2(WT)+$JNK1^{CA}$, PKM2(T365A)+$JNK1^{CA}$, respectively. Data shown are mean±SEM of three independent experiments.

Supplementary Fig. 9. Full western blots
Full-unedited gels for:

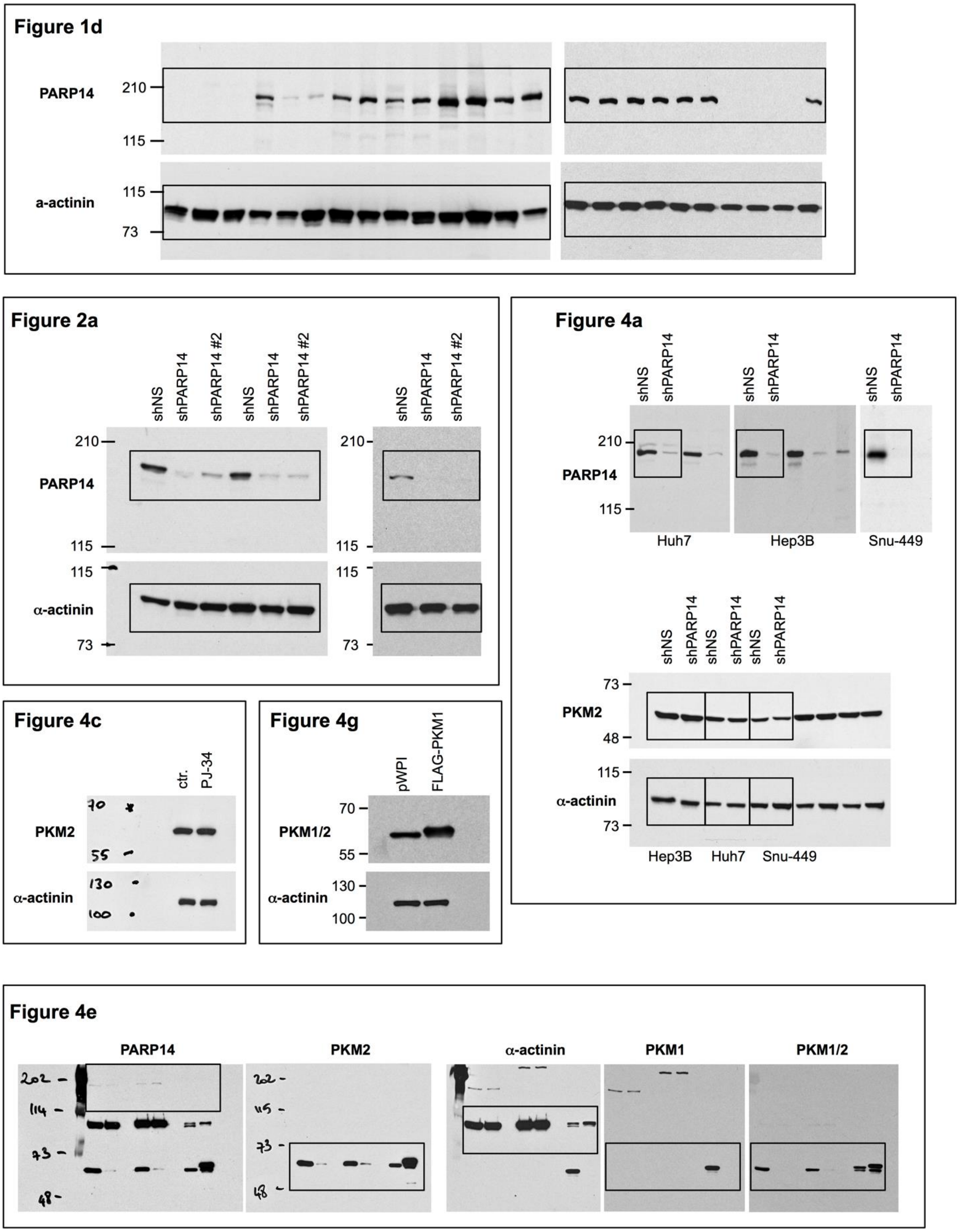

Supplementary Fig. 9 (continued).
Full-unedited gels for:

Figure 5b

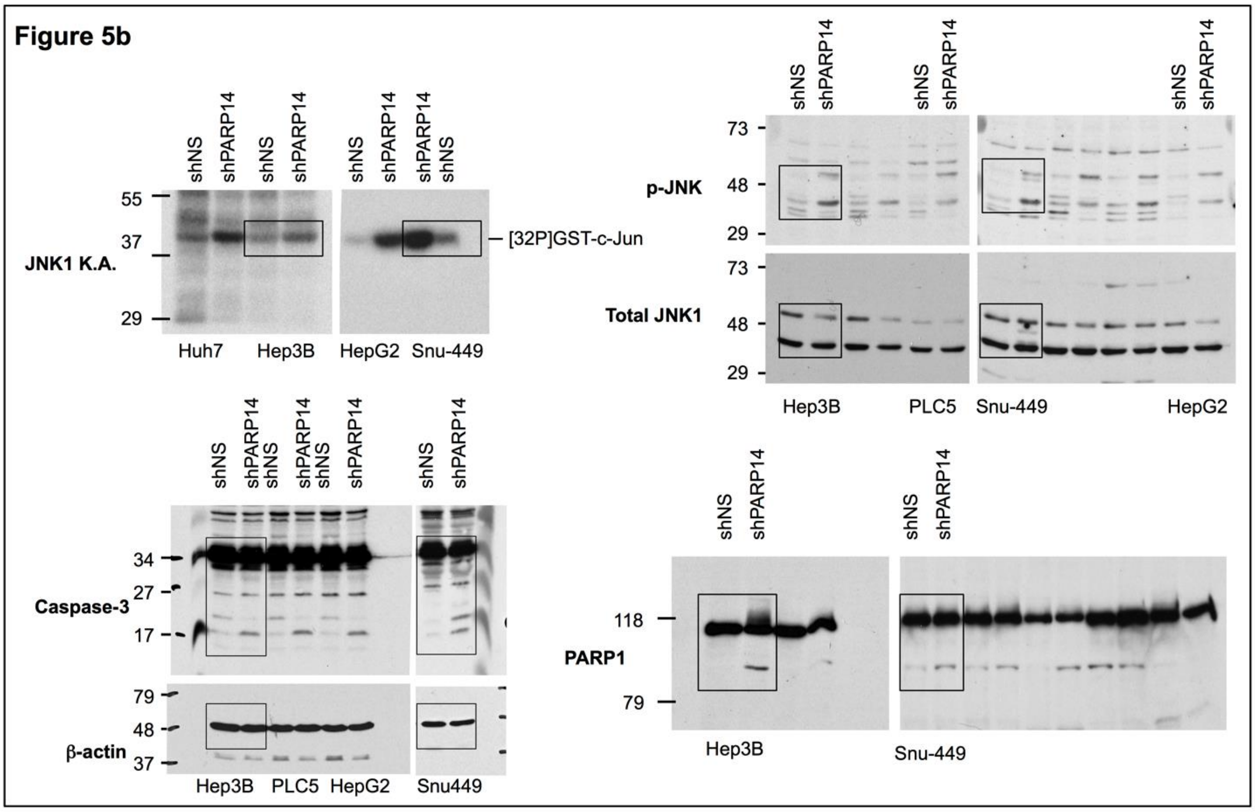

Figure 5b

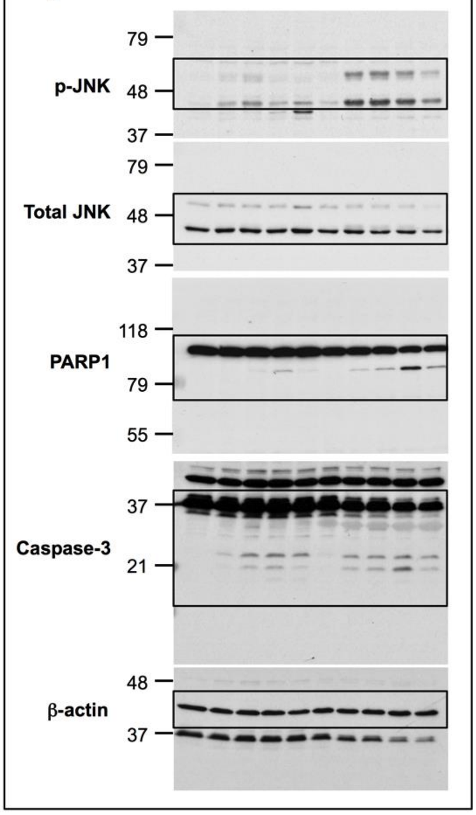

Figure 5f

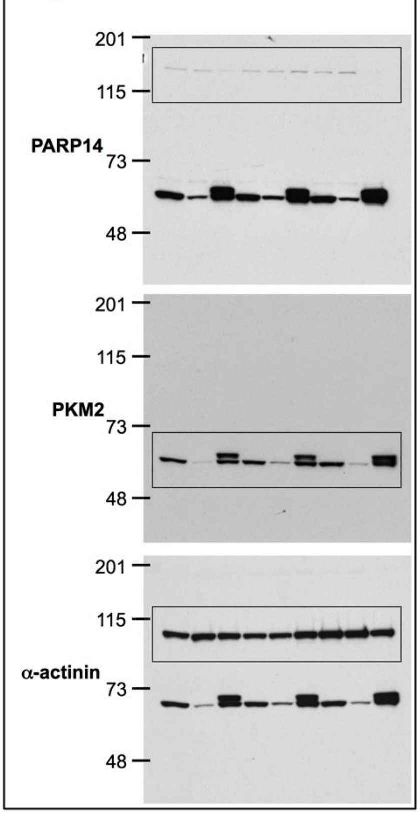

Figure 6a

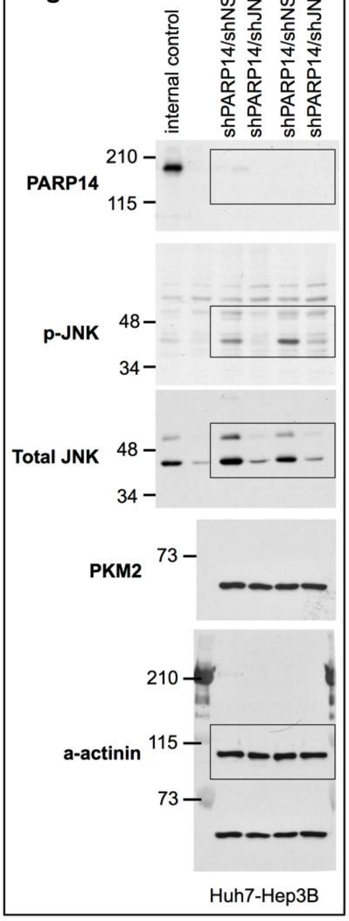

Supplementary Fig. 9 (continued).
Full-unedited gels for:

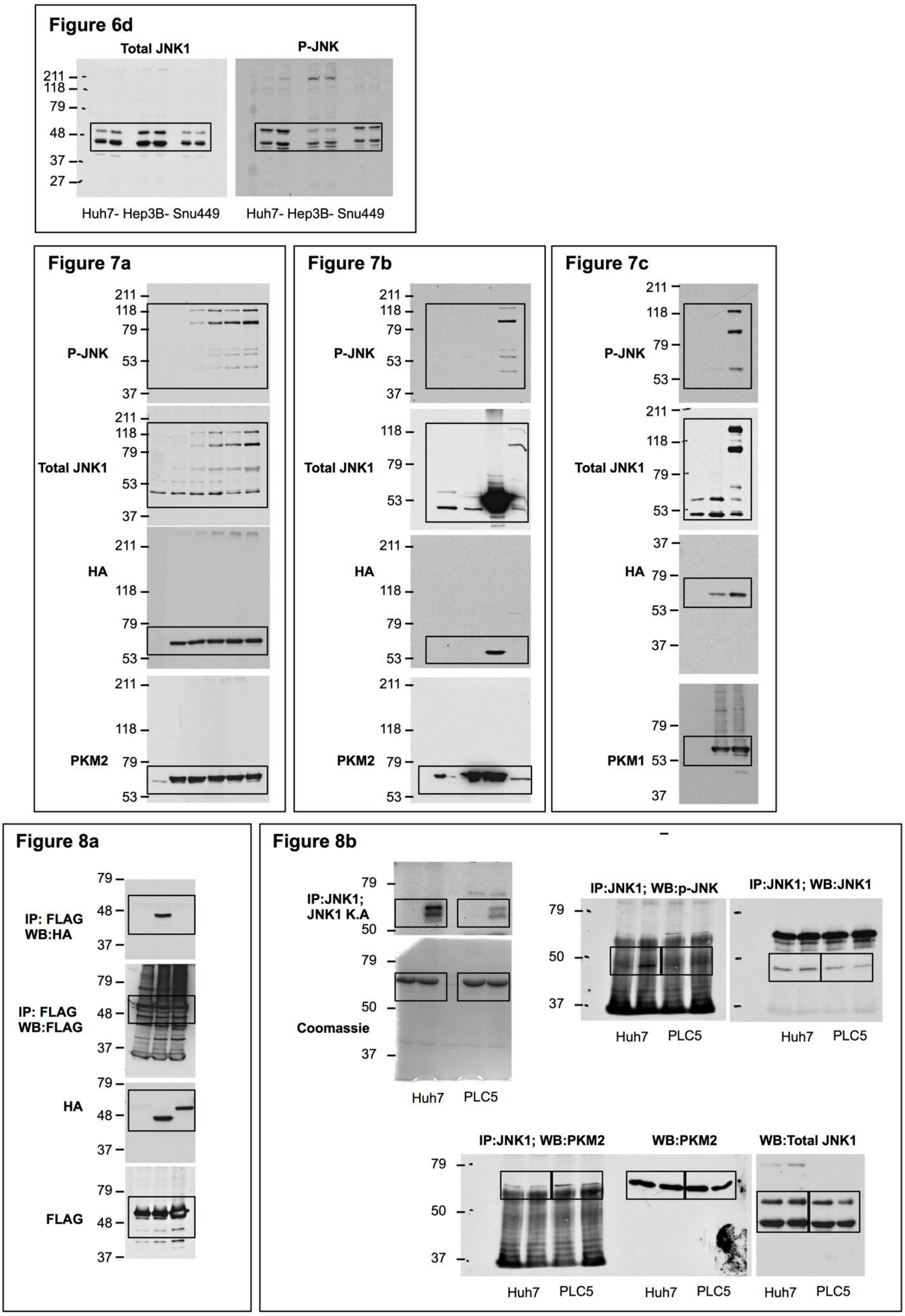

Supplementary Fig. 9 (continued).
Full-unedited gels for:

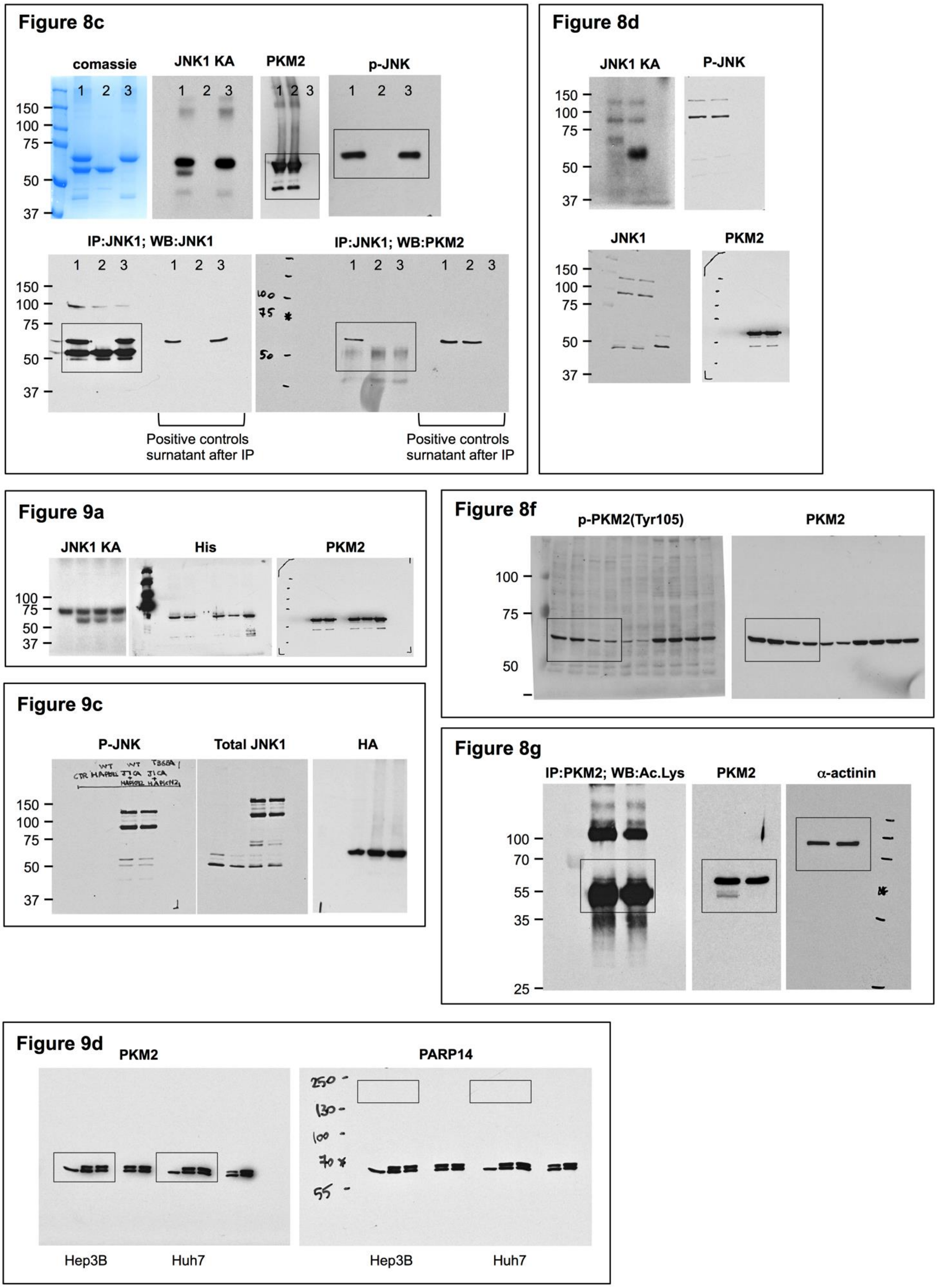

Supplement: Supplementary Information — Supplementary Figures 1-9 [file ncomms8882-s1.pdf]
